# Single-shot quantum error correction with the three-dimensional subsystem toric code

**DOI:** 10.1038/s41467-022-33923-4

**Published:** 2022-10-21

**Authors:** Aleksander Kubica, Michael Vasmer

**Affiliations:** 1 Perimeter Institute for Theoretical Physics, Waterloo, ON N2L 2Y5 Canada; 2 Institute for Quantum Computing, University of Waterloo, Waterloo, ON N2L 3G1 Canada; 3 AWS Center for Quantum Computing, Pasadena, CA 91125 USA; 4 California Institute of Technology, Pasadena, CA 91125 USA

**Keywords:** Quantum information, Computer science, Qubits, Topological matter

## Abstract

Fault-tolerant protocols and quantum error correction (QEC) are essential to building reliable quantum computers from imperfect components that are vulnerable to errors. Optimizing the resource and time overheads needed to implement QEC is one of the most pressing challenges. Here, we introduce a new topological quantum error-correcting code, the three-dimensional subsystem toric code (3D STC). The 3D STC can be realized with geometrically-local parity checks of weight at most three on the cubic lattice with open boundary conditions. We prove that one round of parity-check measurements suffices to perform reliable QEC with the 3D STC even in the presence of measurement errors. We also propose an efficient single-shot QEC decoding strategy for the 3D STC and numerically estimate the resulting storage threshold against independent bit-flip, phase-flip and measurement errors to be *p*_STC_ ≈ 1.045%. Such a high threshold together with local parity-check measurements make the 3D STC particularly appealing for realizing fault-tolerant quantum computing.

## Abstract (web summary)

Topological quantum error correction is a promising approach towards fault-tolerant quantum computing, but suffers from large time overhead. Here, the authors generalise the stabiliser toric code to a single-shot 3D subsystem toric code, featuring good performance and resilience to measurement errors.

## Introduction

Building reliable and scalable universal quantum computers is a heroic endeavor^1–5^, which requires the implementation of fault-tolerant protocols^6–9^. Even the substantially simpler task of storing quantum information is very challenging and requires the usage of quantum error correction (QEC) techniques to detect and eliminate faults. Due to unreliable physical components, QEC is itself a noisy process, which, if carried out haphazardly, can destroy encoded logical information. Nevertheless, QEC together with fault-tolerant gadgets to implement logical gates on the encoded information allow one, in principle, to perform arbitrary long quantum computations provided the noise affecting the system is below some constant threshold value^10–13^. However, questions about the practicality, noise tolerance, and resource requirements for various realizations of universal quantum computation still remain topics of active research^14–20^.

Topological quantum error-correcting codes^21,22^ provide a realistic and resource-efficient approach to building scalable quantum computers. Codes in this class have desirable features, such as efficient classical decoding algorithms with high storage thresholds and fault-tolerant logical gates with low overhead. Importantly, topological quantum codes can be realized by placing qubits on geometrical lattices and measuring only geometrically local parity checks. We emphasize that locality is critical not only from the perspective of fault tolerance but also from the fact that the physical interactions that we can engineer have a local nature. To experimentally realize topological quantum codes, we are restricted to at most three spatial dimensions (unless we allow non-local connections between qubits, which can effectively boost the dimensionality of the system).

The archetypal topological quantum code, the toric code^23,24^, can be engineered in two spatial dimensions. There has been a lot of effort devoted to realizing 2D codes such as the toric code, both from the theory as well as the experimental side^25–32^. In the presence of measurement errors, one way to perform reliable QEC with the toric code is to use a simple fault-tolerant method to extract the syndrome—it suffices to repeat parity-check measurements to gain confidence in their outcomes^14,21,33^ Unfortunately, the number of measurement rounds necessarily grows with the code size, and thus the penalty one pays is the increased time overhead and the need to store measurement outcomes. Subsequently, QEC extends over time, the system effectively becomes (2 + 1)D, and the overall fault-tolerant protocols become more complicated.

Recently, a lot of effort has been devoted to developing QEC techniques that do not require repeated rounds of parity-check measurements in the presence of measurement errors^34–38^. Rather, these techniques rely on a careful choice of which parity checks to measure, as well as on redundancies among the measurement outcomes due to the choice of an overcomplete set of parity checks. Unfortunately, if these techniques are applied to the 2D toric code, then the geometric locality of the system is lost, as we would need to measure some high-weight non-local parity checks, which is a serious limitation.

**Fig. 1:**
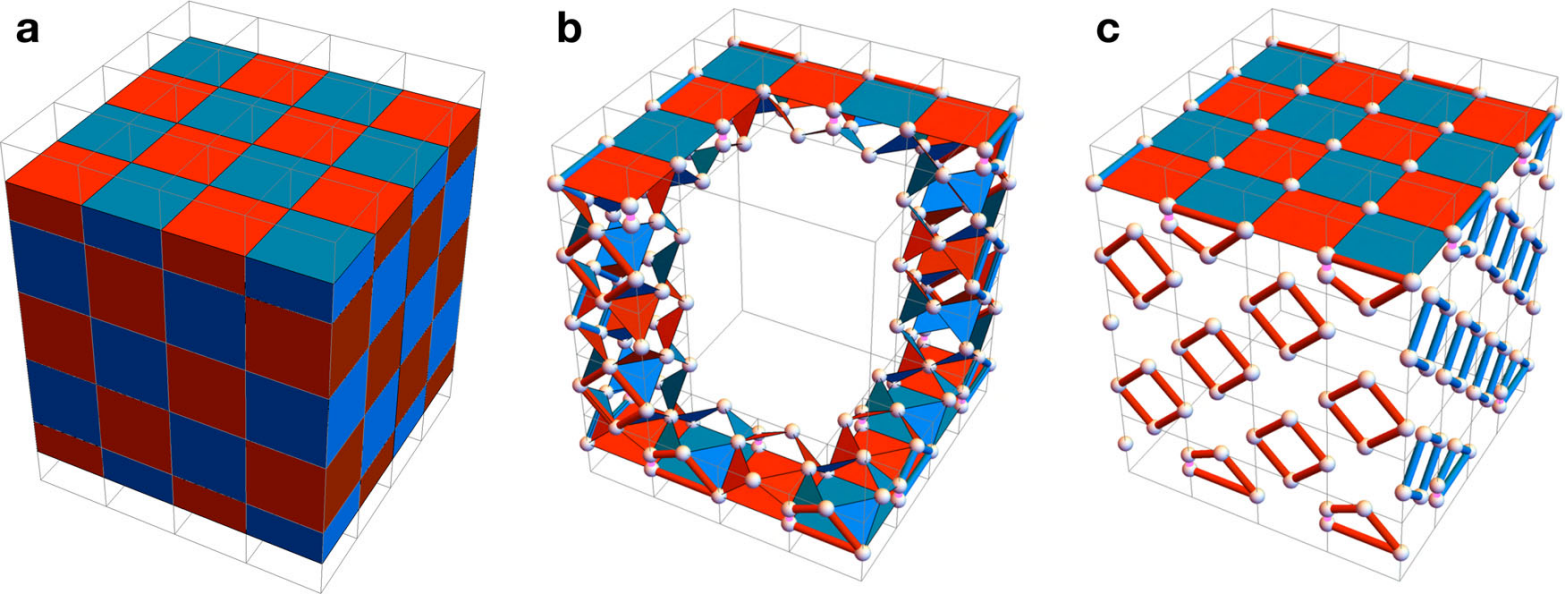
First glance at the 3D STC. **a** The cubic lattice with open boundary conditions and the linear size *L* = 4. Its cubic volumes can be colored in red and blue in a checkerboard pattern. **b**, **c** We define the 3D STC on the lattice ${{{{{{{{\mathcal{L}}}}}}}}}_{{{{{{{{\rm{cub}}}}}}}}}^{*}$ (described in the section “Results”) according to Fig. 2a, c. We illustrate the support of some of the *X*- and *Z*-type gauge operators (red and blue shapes). Qubits are placed on the edges of the cubic lattice (represented by white dots), with additional qubits near the top and bottom boundaries. Pink edges near the top and bottom boundaries represent the weight-two *X*- and *Z*-type gauge operators. Bare and dressed logical Pauli *Z* (Pauli *X*) operators can be supported on, respectively, the front (right) boundary and its intersection with the top boundary, where the front, right, and top boundaries are depicted in **c**.

In this article, we propose a radically different realization of the toric code capable of handling measurement errors, which relies on single-shot QEC discovered by Bombín^39^. Intuitively, single-shot QEC guarantees that one can perform reliable QEC without repeating (geometrically local) parity-check measurements. In order to achieve this, we introduce a subsystem version of the toric code, the three-dimensional subsystem toric code (3D STC). Remarkably, the 3D STC is a topological quantum code that can be realized on the cubic lattice with open boundary conditions and low-weight parity-check measurements (see Fig. 1). Due to its simplicity, the 3D STC can be viewed as the quintessential topological code demonstrating single-shot QEC.

## Results

Our work comprises three parts. First, we introduce the 3D STC model. Then, we propose a single-shot decoding algorithm for the 3D STC and numerically estimate its performance. Lastly, we prove that single-shot QEC is possible with the 3D STC.

### Model

We start this section by presenting a simple and concrete realization of the 3D STC on the lattice ${{{{{{{{\mathcal{L}}}}}}}}}_{{{{{{{{\rm{cub}}}}}}}}}^{*}$, which is based on the cubic lattice with open boundary conditions. The resulting 3D STC has one logical qubit and code distance proportional to the linear size of ${{{{{{{{\mathcal{L}}}}}}}}}_{{{{{{{{\rm{cub}}}}}}}}}^{*}$. We note that the 3D STC is more general than the realization detailed in this section; see the section “Methods” and Supplementary Note 3.

A subsystem code is a generalization of a stabilizer code. Intuitively, a subsystem code is like a stabilizer code, except we only encode quantum information into a subset of the qubits in the stabilizer subspace. We refer to the encoded qubits in this subset as the logical qubits; the remaining qubits are called the gauge qubits. A subsystem code is specified by its gauge group ${{{{{{{\mathcal{G}}}}}}}}$, which is a subgroup of the Pauli group ${{{{{{{\mathcal{P}}}}}}}}$ that may contain −*I*. We note that when ${{{{{{{\mathcal{G}}}}}}}}$ is non-Abelian, it necessarily contains −*I*. Let ${{{{{{{\mathcal{Z}}}}}}}}({{{{{{{\mathcal{G}}}}}}}})$ denote the centralizer of ${{{{{{{\mathcal{G}}}}}}}}$ in the Pauli group ${{{{{{{\mathcal{P}}}}}}}}$, i.e., all the elements in ${{{{{{{\mathcal{P}}}}}}}}$ that commute with every element in ${{{{{{{\mathcal{G}}}}}}}}$. Ignoring the phases, we refer to the center of the gauge group ${{{{{{{\mathcal{G}}}}}}}}$ as the stabilizer group ${{{{{{{\mathcal{S}}}}}}}}$, i.e., ${{{{{{{\mathcal{S}}}}}}}}=({{{{{{{\mathcal{Z}}}}}}}}({{{{{{{\mathcal{G}}}}}}}})\cap {{{{{{{\mathcal{G}}}}}}}})/\langle i\rangle$. Whenever the gauge group ${{{{{{{\mathcal{G}}}}}}}}$ does not contain −*I*, it can be viewed as the stabilizer group defining a stabilizer code. Lastly, we say that a stabilizer or a subsystem code is a CSS code^40,41^ if its generators can be chosen as either Pauli *X* or *Z* operators.

**Fig. 2:**
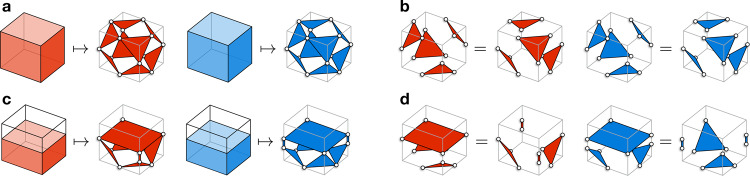
The gauge operators and stabilizers of the 3D STC. **a** The bulk (weight-three) *X*- and *Z*-type gauge operators, where we depict the support of operators as red and blue triangles, respectively. **b** The bulk (weight-12) *X*- and *Z*-type stabilizer operators, which can be formed in two different ways by multiplying gauge operators. **c** The boundary *X*- and *Z*-type gauge operators. **d** The boundary (weight-10) *X*- and *Z*-type stabilizer operators, which can be formed in two different ways by multiplying gauge operators.

The 3D STC is a topological quantum code and is also a CSS subsystem code. We start with a simple and concrete realization of the 3D STC on the lattice ${{{{{{{{\mathcal{L}}}}}}}}}_{{{{{{{{\rm{cub}}}}}}}}}^{*}$, which is based on the cubic lattice with open boundary conditions (see Fig. 1a). The cubic volumes of the cubic lattice can be colored in red and blue in a checkerboard pattern. In the bulk of the lattice, we place one qubit on every edge. For every red and blue volume we introduce eight weight-three *X*- and *Z*-type gauge operators, respectively, as shown in Fig. 2a. Then, weight-12 *X*- and *Z*-type stabilizer operators, which we associate with red and blue volumes, can be formed in two different ways by multiplying gauge operators from Fig. 2a, as shown in Fig. 2b. Near the top boundary of the lattice, we place one additional qubit on every other vertical edge and introduce seven *X*- and *Z*-type gauge operators for every red and blue volume as shown in Fig. 2c. Similarly, weight-10 *X*- and *Z*-type stabilizer operators can be formed in two different ways by multiplying gauge operators from Fig. 2c as shown in Fig. 2d. Lastly, on every outward-facing side of a blue (respectively, red) volume along the front (right) boundary of the lattice, we introduce four weight-two *X*-type (*Z*-type) gauge operators, which we can further multiply in two different ways to form a weight-four *X*-type (*Z*-type) stabilizer operator. We remark that the bottom, rear and left boundaries are the same as the top, front and right boundaries, respectively, as the lattice is invariant under point reflection through its center. We illustrate some of the gauge generators of the resulting 3D STC on the lattice ${{{{{{{{\mathcal{L}}}}}}}}}_{{{{{{{{\rm{cub}}}}}}}}}^{*}$ in Fig. 1b, c. Throughout the article, we use red and blue to depict the support of Pauli *X* and *Z* operators, respectively.

In order to reduce the spread of errors, one may want to reduce the weight of the weight four gauge operators on the top and bottom boundaries. In Supplementary Note 1, we show that the gauge group of the 3D STC defined on ${{{{{{{{\mathcal{L}}}}}}}}}_{{{{{{{{\rm{cub}}}}}}}}}^{*}$ can be modified such that all gauge operators have weight at most three, at the cost of introducing some additional ancilla qubits.

The bare and dressed logical Pauli operators of the subsystem code ${{{{{{{\mathcal{G}}}}}}}}$ are defined as the elements of ${{{{{{{\mathcal{Z}}}}}}}}({{{{{{{\mathcal{G}}}}}}}})$ and ${{{{{{{\mathcal{Z}}}}}}}}({{{{{{{\mathcal{S}}}}}}}})$, respectively. Dressed logical operators, unlike bare logical operators, may change the state of gauge qubits, however, no logical information is encoded into them. Similarly to the logical operators of the 3D stabilizer toric code, bare and dressed logical Pauli operators of the 3D STC can be expressed as 2D sheet-like and 1D string-like operators connecting opposite boundaries of the lattice ${{{{{{{{\mathcal{L}}}}}}}}}_{{{{{{{{\rm{cub}}}}}}}}}^{*}$. For instance, bare logical Pauli *Z* and *X* operators can be fully supported within, respectively, the front or rear and left or right side boundaries (see Fig. 1c).

If the linear size of the lattice ${{{{{{{{\mathcal{L}}}}}}}}}_{{{{{{{{\rm{cub}}}}}}}}}^{*}$ is *L*, then the code parameters of the 3D STC on ${{{{{{{{\mathcal{L}}}}}}}}}_{{{{{{{{\rm{cub}}}}}}}}}^{*}$ are

1 $$[\![N=3{L}^{3}+6{L}^{2}+5L+1,\,K=1,\,D=L+1]\!],$$

where *N* and *K* are the numbers of physical and logical qubits, and *D* is the code distance (defined as the weight of the smallest dressed logical operator). The number of logical qubits of the 3D STC defined on ${{{{{{{{\mathcal{L}}}}}}}}}_{{{{{{{{\rm{cub}}}}}}}}}^{*}$ can be derived from the general method for constructing the 3D STC presented in the section “Methods”.

Note that in the case of biased noise we may optimize the region where we define the 3D STC. In particular, we can use a cuboidal region of size *L*_*X*_ × *L*_*Z*_ × *L*_*M*_, where L_*_ is, roughly speaking, proportional to $-{(\log {p}_{*})}^{-1}$, and *p*_*X*_, *p*_*Z*_ and *p*_*M*_ are the error rates for Pauli *X*, Pauli *Z*, and measurement errors, respectively.

**Fig. 3:**
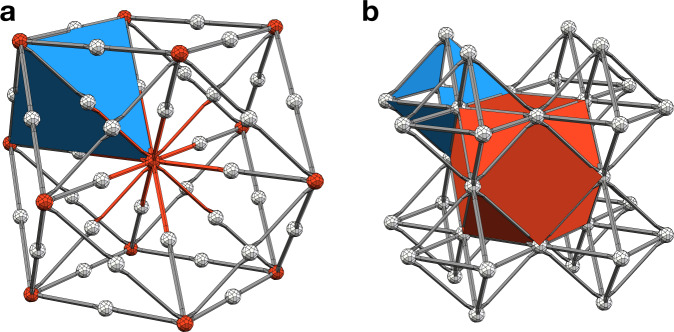
Two equivalent definitions of the 3D stabilizer toric code. **a** The standard construction on a lattice ${{{{{{{\mathcal{K}}}}}}}}$. Qubits (white balls) are placed on the edges, and *X*- and *Z*-stabilizers are identified with vertices (red) and faces (blue). **b** A rectified lattice ${{{{{{{{\mathcal{K}}}}}}}}}^{{{{{{{{\rm{rec}}}}}}}}}$ obtained from ${{{{{{{\mathcal{K}}}}}}}}$. Qubits are placed at vertices (white balls) and *X*- and *Z*-stabilizers are identified with volumes (red) and faces (blue).

To understand how the 3D STC is related to the 3D stabilizer toric code, it is useful to recast the description of the latter. The standard way of defining the 3D toric code on a 3D lattice ${{{{{{{\mathcal{K}}}}}}}}$ is to place qubits at every edge of ${{{{{{{\mathcal{K}}}}}}}}$ and to introduce *X*- and *Z*-type stabilizer generators for every vertex *v* and face *f* of ${{{{{{{\mathcal{K}}}}}}}}$ as the product of Pauli *X* and *Z* operators on qubits adjacent to *v* and *f* (see Fig. 3a). However, there is an equivalent way to describe the 3D toric code in the rectified lattice picture^42^, which is similar in spirit to the definition of the color code. Roughly speaking, to obtain a rectified lattice ${{{{{{{{\mathcal{K}}}}}}}}}^{{{{{{{{\rm{rec}}}}}}}}}$ from the lattice ${{{{{{{\mathcal{K}}}}}}}}$ we inflate every vertex of ${{{{{{{\mathcal{K}}}}}}}}$ by introducing an extra volume there (see Fig. 3b). In the rectified lattice picture qubits are placed on vertices, *X*-type stabilizer generators are associated with a subset of volumes and *Z*-type stabilizer generators are associated with a subset of faces.

By comparing Fig. 3b with Fig. 2a, b, we see that the *X*-type stabilizer generators and *Z*-type gauge generators of the 3D STC are exactly the *X*- and *Z*-type stabilizer generators of a 3D stabilizer toric code (albeit defined on an unusual lattice, the tetrahedral–octahedral lattice). Therefore we have the relation

2 $${{{{{{{\mathcal{S}}}}}}}}\le {{{{{{{{\mathcal{S}}}}}}}}}_{{{{{{{{\rm{3DST}}}}}}}}}\le {{{{{{{\mathcal{G}}}}}}}},$$

where ${{{{{{{\mathcal{S}}}}}}}}$ and ${{{{{{{\mathcal{G}}}}}}}}$ are the stabilizer and gauge groups of the 3D STC defined on ${{{{{{{{\mathcal{L}}}}}}}}}_{{{{{{{{\rm{cub}}}}}}}}}^{*}$ and ${{{{{{{{\mathcal{S}}}}}}}}}_{{{{{{{{\rm{3DST}}}}}}}}}$ is the stabilizer group of the 3D stabilizer toric code defined on ${{{{{{{{\mathcal{L}}}}}}}}}_{{{{{{{{\rm{cub}}}}}}}}}^{*}$ (with *X* stabilizer generators associated with red volumes). Thus, a state in the code space of the 3D toric code is also in the code space of the 3D STC, with its gauge qubits in a certain determined state. Furthermore, using the procedure of gauge fixing we can ensure that all *Z*-type gauge generators in ${{{{{{{\mathcal{G}}}}}}}}$ are satisfied, thereby fixing the gauge qubits of a state in the code space of the 3D STC such that the state is also in the code space of the 3D stabilizer toric code.

### Single-shot decoding

Active QEC comprises the detection and correction of errors. For stabilizer and subsystem codes, we first measure parity checks corresponding to some stabilizer and gauge operators, respectively. Then, using the obtained classical information we infer the stabilizer syndrome, i.e., the set of all stabilizers returning −1 measurement outcome. We emphasize that in order to reliably infer the stabilizer syndrome in the presence of measurement errors one may have to repeat parity-check measurements, which in turn results in a significant time overhead, as exemplified by the 2D toric code. Lastly, we use classical decoding algorithms and find an appropriate recovery for the given stabilizer syndrome.

Performing optimal QEC for generic stabilizer codes is a computationally hard task even in the absence of measurement errors^43^. However, for topological quantum codes there exist various decoding algorithms with good performance, many of which rely on solving the minimum-weight perfect-matching (MWPM) problem^21,44–46^. The MWPM problem, roughly speaking, is the task of pairing some subset of the vertices of a given graph, which, importantly, can be solved efficiently^47^.

An efficient QEC strategy for the 3D STC, which we propose, requires only one round of parity-check measurements and works reliably even in the presence of measurement errors. Our QEC strategy, which we name the single-shot MWPM decoder, consists of two steps: (i) syndrome estimation and (ii) ideal MWPM decoding. Remarkably, both steps can be reduced to the MWPM problem, which should be contrasted with alternative approaches to single-shot QEC^48–52^. We numerically benchmark the performance of the single-shot MWPM decoder against bit-flip and phase-flip noise in the presence of measurement errors (phenomenological noise) and estimate the storage threshold to be *p*_STC_ ≈ 1.045%; see the Methods section. We provide further details on our decoder and numerical results in the section “Methods”.

### Proof of single-shot QEC

Local operations play a central role in fault-tolerant quantum computation, as they preserve the local structure of noise, and thus are trivially fault-tolerant. However, strictly local operations, such as transversal gates or cellular-automata decoders, are limited—the computational power of the former is restricted^53–62^, whereas the latter requires that the syndrome has some underlying structure^63–66^.

One way to avoid the aforementioned limitations is to consider quantum-local operations^39^, i.e., local operations that depend on classical information stored only for a limited time. Such processes are physically motivated, as quantum operations are typically constrained by geometrically local interactions, whereas classical information can be processed globally in a reliable way. Examples of quantum-local operations include the procedure of gauge fixing^67,68^ and the single-shot MWPM decoder, which allow for, respectively, a fault-tolerant universal gate set without magic state distillation and single-shot QEC. Unfortunately, quantum-local operations are not a priori fault-tolerant, as they are not guaranteed to preserve the local structure of noise.

In Supplementary Note 4, we prove that the single-shot MWPM decoder for the 3D STC is indeed fault-tolerant. In other words, performing repeated rounds of error correction in the presence of measurement errors will not lead to the uncontrollable accumulation of errors and the logical information encoded in the 3D STC will be protected for a long time. This, in turn, rigorously establishes that the 3D STC allows for QEC in a single-shot manner, which drastically reduces the time overhead associated with QEC.

## Discussion

In our work we: (i) introduced a new topological quantum code, the 3D STC, which is a subsystem version of the toric code, (ii) developed a single-shot decoding algorithm for the 3D STC and numerically estimated its performance, and (iii) proved that single-shot QEC is possible with the 3D STC. We believe that the 3D STC provides the canonical example of a topological quantum code demonstrating single-shot QEC.

Topological quantum error-correcting codes are alluring not only from the perspective of QEC but also from the perspective of quantum many-body physics. Even the simplest topological quantum codes, which belong to the class of stabilizer codes^69^ or their slight generalization, subsystem codes^70^, illustrate a variety of physical concepts. For instance, the ground state of the 2D toric code is an epitome of a topologically ordered state. In three dimensions, Chamon’s model^71^ and the cubic code^72^ provide concrete realizations of exotic quantum phases of matter with fractal-like excitations. In four dimensions, the 4D toric code gives rise to a local commuting Hamiltonian that exhibits the phenomenon of self-correction^73,74^. Analogously, the 3D STC demonstrates single-shot QEC.

The 3D STC is similar to another topological quantum code, the 3D gauge color code^75^. The 3D STC and 3D gauge color code constitute subsystem versions of the stabilizer toric code and the stabilizer color code^76–78^, respectively. Both codes facilitate single-shot QEC, as well as a fault-tolerant universal gate set without magic state distillation^75,79^. However, the 3D STC is more appealing due to its simplicity—it can be realized on the cubic lattice with open boundary conditions by measuring geometrically local parity checks of weight at most three. This should be contrasted with the known realizations of the 3D gauge color code, which require parity checks of weight at least six. In addition, for the same error model as we consider in the section “Methods”, the storage threshold of the gauge color code has been estimated to be ~0.31%, approximately three times smaller than the 3D STC storage threshold. Subsequently, the 3D STC provides significantly better protection from errors than the 3D gauge color code^48,80^.

The parity checks of the 3D STC are weight 3, so we expect one can find short-depth syndrome extraction circuits and that errors will not propagate badly in these circuits. Therefore, we anticipate that the circuit-level storage threshold of the 3D STC will not be reduced too much when compared with the phenomenological storage threshold, and may even be comparable with the circuit-level storage threshold of the 2D toric code (0.5–1.1% depending on the error model^81^).

Despite the close connection between the toric code and the color code in *d* ≥ 2 dimensions^82^, a genuine subsystem generalization of the toric code was not known. The 3D STC provides such a generalization. Although in the main text we focus on the three-dimensional case, our construction is more general and works in any dimension *d* ≥ 3 (see Supplementary Note 3 for details). Such higher-dimensional constructions are particularly appealing from the perspective of realizing self-correction and fault-tolerant non-Clifford logical gates^82–84^. We remark that in the special case of two dimensions there exist realizations of the toric code as a subsystem code^85,86^. However, by applying constant-depth circuits composed of geometrically local gates one can remove the gauge qubits and effectively map those models to the stabilizer toric code. This, however, is not possible for the 3D STC, and therefore the 3D STC is fundamentally different from the 3D toric code.

The 3D STC can be used to implement a fault-tolerant universal gate set {*H*, *T*, CNOT} without state distillation. Namely, we can consider a version of the 3D STC supported within a tetrahedral region, which has different transversal logical operations. Since the 3D STC is a CSS code, it has the transversal CNOT gate. Moreover, depending on the state of the gauge qubits, it has either the transversal Hadamard gate *H* or the transversal *T* = diag(1, e^i*π*/4^) gate^87^.

While 3D codes have advantages over 2D codes such as single-shot error correction, there are also some disadvantages that come with using 3D codes. First, although the spacetime cost of single-shot error correction using 3D codes scales in the same way as the spacetime cost of *d* rounds of error correction using 2D codes, the space cost (the number of qubits) required in the 3D case is greater. One can therefore view single-shot error correction as using additional qubits to reduce the time overhead of error correction, while also providing resilience against time-correlated noise^88^. Second, the 3D connectivity required to implement 3D codes makes them difficult to engineer in architectures with planar connectivity. However, there has recently been significant progress in developing architectures with beyond-planar connectivity^89–92^, making 3D codes a more realistic prospect.

Lastly, we remark that the concepts of self-correction and single-shot QEC are closely connected. Typically, the former implies the latter, as exemplified by the 4D toric code, however, the converse is not immediate. Thus, one fundamental problem worth exploring is whether the 3D STC can give rise to a self-correcting topological phase. We expect that by taking some local gauge operators of the 3D STC we can construct Hamiltonians that possess symmetry-protected topological order and thermal stability in the presence of 1-form symmetries, analogously to the Hamiltonians arising from the 3D gauge color code^93–95^.

## Methods

### Model

In this section, we discuss a systematic construction of the 3D STC that goes beyond the cubic lattice. We restrict our attention to octahedral lattices, which are obtained by gluing finitely many octahedra together along their proper faces of matching dimensions. An octahedral lattice ${{{{{{{\mathcal{L}}}}}}}}$ is a collection of vertices, edges, faces, and volumes, but it can be also viewed as a topological space. In particular, ${{{{{{{\mathcal{L}}}}}}}}$ is a manifold, possibly with a boundary.

**Fig. 4:**
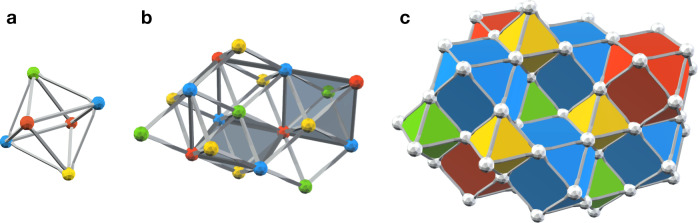
Colorable octahedral lattice examples. **a** An antipodally colored octahedron. **b** A colorable octahedral lattice ${{{{{{{\mathcal{L}}}}}}}}$. We obtain ${{{{{{{\mathcal{L}}}}}}}}$ from the body-centered cubic lattice by first assigning colors *G* or *Y* to the vertices at the centers of the cubic volumes, and colors *R* or *B* to all other vertices, followed by filling in octahedra. We only depict two octahedra (shaded in gray). **c** The dual lattice ${{{{{{{{\mathcal{L}}}}}}}}}^{*}$ is obtained from ${{{{{{{\mathcal{L}}}}}}}}$ by replacing its volumes, faces, edges, and vertices by vertices, edges, faces, and volumes, respectively. The lattice ${{{{{{{{\mathcal{L}}}}}}}}}_{{{{{{{{\rm{cub}}}}}}}}}^{*}$ from the section “Results” is a simplified version of ${{{{{{{{\mathcal{L}}}}}}}}}^{*}$, where the *G* and *Y* volumes are omitted. Note that the 3D STC can be defined on ${{{{{{{{\mathcal{L}}}}}}}}}^{*}$ by placing one qubit on every vertex, and introducing *X*- and *Z*-type gauge generators for every *RG* or *RY* face and *BG* or *BY* face, respectively.

We say that an octahedral volume is antipodally colored if the three pairs of its opposite vertices are two *R* vertices, two *B* vertices, and *G* and *Y* vertices (see Fig. 4a). Then, we say that an octahedral lattice is colorable if we can assign four colors *R*, *B*, *G* and *Y* to its vertices in such a way that every octahedral volume is antipodally colored. Note that other cells of the octahedral lattice inherit colors in a natural way. For example, an edge between *R* and *G* vertices has the color *RG*. In Fig. 4, we illustrate an example of a colorable octahedral lattice ${{{{{{{\mathcal{L}}}}}}}}$ as well as its dual lattice ${{{{{{{{\mathcal{L}}}}}}}}}^{*}$. We also remark that the lattice dual to the lattice ${{{{{{{{\mathcal{L}}}}}}}}}_{{{{{{{{\rm{cub}}}}}}}}}^{*}$ from the section “Results” forms a colorable octahedral lattice.

Let ${{{{{{{\mathcal{L}}}}}}}}$ be a three-dimensional lattice, which satisfies the following two conditions:

(i) ${{{{{{{\mathcal{L}}}}}}}}$ is an octahedral lattice,
(ii) ${{{{{{{\mathcal{L}}}}}}}}$ is colorable.

We use a notation ${{{{{{{{\mathcal{L}}}}}}}}}_{i}$ to denote the set of all *i*-dimensional cells of ${{{{{{{\mathcal{L}}}}}}}}$, and write ${{{{{{{{\mathcal{L}}}}}}}}}_{i}^{C}$ to further restrict our attention to *i*-dimensional cells of color *C*.

**Fig. 5:**
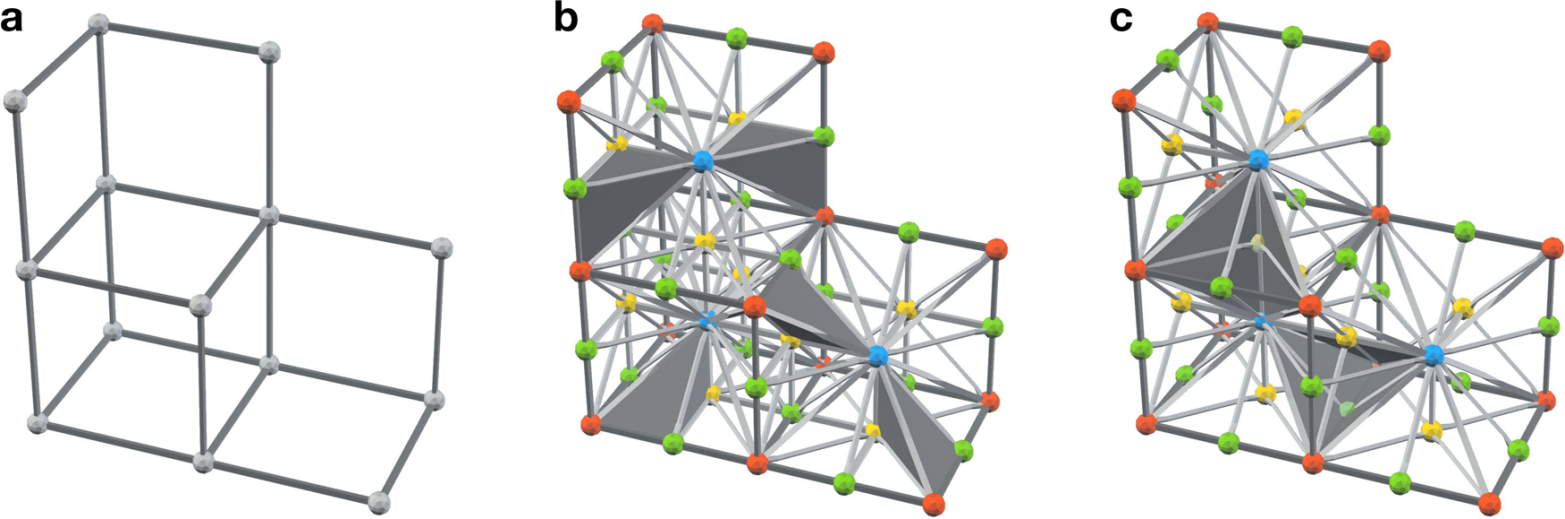
A procedure for constructing a colorable octahedral lattice ${{{{{{{{\mathcal{J}}}}}}}}}^{{{{{{{{\rm{oct}}}}}}}}}$. **a** We start with an arbitrary three-dimensional lattice ${{{{{{{\mathcal{J}}}}}}}}$ without boundary. **b** We convert ${{{{{{{\mathcal{J}}}}}}}}$ into a simplicial complex ${{{{{{{{\mathcal{J}}}}}}}}}^{{{{{{{{\rm{sim}}}}}}}}}$. The vertices of ${{{{{{{{\mathcal{J}}}}}}}}}^{{{{{{{{\rm{sim}}}}}}}}}$ can be colored in *R*, *G*, *Y* and *B*, since they correspond to the vertices, edges, faces and volumes of ${{{{{{{\mathcal{J}}}}}}}}$. We shade some tetrahedra in gray. **c** We obtain ${{{{{{{{\mathcal{J}}}}}}}}}^{{{{{{{{\rm{oct}}}}}}}}}$ by finding for every *GY* edge in ${{{{{{{{\mathcal{J}}}}}}}}}_{3}^{{{{{{{{\rm{sim}}}}}}}}}$ four tetrahedra containing it and merging them into a single cell. We shade some octahedral volumes in gray.

One can transform any three-dimensional lattice ${{{{{{{\mathcal{J}}}}}}}}$ without boundary into a colorable octahedral lattice ${{{{{{{{\mathcal{J}}}}}}}}}^{{{{{{{{\rm{oct}}}}}}}}}$. In the first step, we convert ${{{{{{{\mathcal{J}}}}}}}}$ into a simplicial *d*-complex ${{{{{{{{\mathcal{J}}}}}}}}}^{{{{{{{{\rm{sim}}}}}}}}}$, where *d* denotes the dimensionality of ${{{{{{{\mathcal{J}}}}}}}}$. This step is general and does not require that *d* = 3. For each flag of ${{{{{{{\mathcal{J}}}}}}}}$, we include a corresponding *d*-simplex in ${{{{{{{{\mathcal{J}}}}}}}}}^{{{{{{{{\rm{sim}}}}}}}}}$. We recall that a (geometrical) flag is a sequence of cells of ${{{{{{{\mathcal{J}}}}}}}}$, where each cell is contained in the next and there is exactly one *i*-cell of each dimension *i* ∈ {0, …, *d*}. We note that the faces of a given *d*-simplex correspond to the non-empty subsets of its corresponding flag. In particular, the vertices of a given *d*-simplex correspond to the individual *i*-cells contained in its corresponding flag. In the second step, since the vertices of ${{{{{{{{\mathcal{J}}}}}}}}}^{{{{{{{{\rm{sim}}}}}}}}}$ correspond to the vertices, edges, faces, and volumes of ${{{{{{{\mathcal{J}}}}}}}}$, we can color them in *R*, *G*, *Y*, and *B*, respectively. We remark that for our transformation to work the vertices could also be colored *G*, *R*, *Y*, and *B*, or *R*, *G*, *B* and *Y*. Then, for every *GY* edge in ${{{{{{{{\mathcal{J}}}}}}}}}_{1}^{{{{{{{{\rm{sim}}}}}}}}}$ we find four tetrahedra in ${{{{{{{{\mathcal{J}}}}}}}}}_{3}^{{{{{{{{\rm{sim}}}}}}}}}$ containing it, and merge them into a single octahedral volume. Note that this step removes all the edges and faces of color *GY* and *RGY* or *BGY*, respectively. It is easy to see that the resulting lattice ${{{{{{{{\mathcal{J}}}}}}}}}^{{{{{{{{\rm{oct}}}}}}}}}$ forms a colorable octahedral lattice. In Fig. 5 we illustrate the process of converting ${{{{{{{\mathcal{J}}}}}}}}$ into ${{{{{{{{\mathcal{J}}}}}}}}}^{{{{{{{{\rm{oct}}}}}}}}}$. We remark that the first step is equivalent to the inflation procedure^77^ presented from the perspective of the dual lattice ${{{{{{{{\mathcal{J}}}}}}}}}^{*}$.

Now we are ready to present a general construction of the 3D STC, which, contrasted with the initial construction from the section “Results”, allows us to define the 3D STC on lattices other than the cubic lattice. Moreover, this construction is not only useful in calculating the number of logical qubits of the 3D STC but also leads to a succinct description of the boundaries of the 3D STC lattice.

Let ${{{{{{{\mathcal{L}}}}}}}}$ be a colorable octahedral lattice. For simplicity, we assume that the lattice ${{{{{{{\mathcal{L}}}}}}}}$ is obtained by tessellating a three-sphere. We identify each octahedral volume of ${{{{{{{\mathcal{L}}}}}}}}$ with a qubit and thus the number of physical qubits is

3 $$N=|{{{{{{{{\mathcal{L}}}}}}}}}_{3}|.$$

For any *i*-dimensional cell, $\delta \in {{{{{{{{\mathcal{L}}}}}}}}}_{i}$ we denote by ${{{{{{{\mathcal{Q}}}}}}}}(\delta )$ the set of all the qubits on octahedra containing *δ*, i.e.,

4 $${{{{{{{\mathcal{Q}}}}}}}}(\delta )=\{\omega \in {{{{{{{{\mathcal{L}}}}}}}}}_{3}|\omega \supseteq \delta \}.$$

By saying that an operator is supported on *δ* we mean that it is supported on the set of qubits ${{{{{{{\mathcal{Q}}}}}}}}(\delta )$ and, for instance, write $X(\delta )={\prod }_{\omega \in {{{{{{{\mathcal{Q}}}}}}}}(\delta )}{X}_{\omega }$, where *X*_*ω*_ denotes Pauli *X* operator acting on qubit *ω*.

The gauge group ${{{{{{{\mathcal{G}}}}}}}}$ of the 3D STC is generated by *X*- and *Z*-type gauge generators supported on, respectively, *RG* or *RY* edges, and *BG* or *BY* edges, namely

5 $${{{{{{{\mathcal{G}}}}}}}}=\left\langle \right.X(\mu ),\,Z(\nu )\left|\right.\mu \in {{{{{{{{\mathcal{L}}}}}}}}}_{1}^{RG}\cup {{{{{{{{\mathcal{L}}}}}}}}}_{1}^{RY},\,\nu \in {{{{{{{{\mathcal{L}}}}}}}}}_{1}^{BG}\cup {{{{{{{{\mathcal{L}}}}}}}}}_{1}^{BY}\rangle .$$

The stabilizer group of the 3D STC is generated by *X*- and *Z*-type stabilizer generators supported on *R* and *B* vertices, i.e.,

6 $${{{{{{{\mathcal{S}}}}}}}}=\left\langle \right.X(u),\,Z(v)\left|\right.u\in {{{{{{{{\mathcal{L}}}}}}}}}_{0}^{R},\,v\in {{{{{{{{\mathcal{L}}}}}}}}}_{0}^{B}\rangle .$$

Indeed, each vertex in ${{{{{{{{\mathcal{L}}}}}}}}}_{0}^{R}\cup {{{{{{{{\mathcal{L}}}}}}}}}_{0}^{B}$ a corresponding stabilizer generator can be formed in two ways by multiplying gauge generators supported on edges incident to that vertex, namely

7 $$X(u)=\mathop{\prod}\limits_{\begin{array}{c}\mu \in {{{{{{{{\mathcal{L}}}}}}}}}_{1}^{RG}:\mu \supset u\end{array}}X(\mu )=\mathop{\prod}\limits_{\begin{array}{c}\mu \in {{{{{{{{\mathcal{L}}}}}}}}}_{1}^{RY}:\mu \supset u\end{array}}X(\mu ),$$

8 $$Z(v)=\mathop{\prod}\limits_{\begin{array}{c}\nu \in {{{{{{{{\mathcal{L}}}}}}}}}_{1}^{BG}:\nu \supset v\end{array}}Z(\nu )=\mathop{\prod}\limits_{\begin{array}{c}\nu \in {{{{{{{{\mathcal{L}}}}}}}}}_{1}^{BY}:\nu \supset v\end{array}}Z(\nu ).$$

To see that stabilizer operators commute with each other and with gauge operators, it suffices to show that a stabilizer generator *X*(*u*) and a gauge generator *Z*(*ν*) commute; the argument for a stabilizer generator *Z*(*v*) and a gauge generator *X*(*μ*) is the same. If the intersection ${{{{{{{\mathcal{Q}}}}}}}}(u)\cap {{{{{{{\mathcal{Q}}}}}}}}(\nu )$ is non-empty, then there exists an octahedral volume $\omega \in {{{{{{{{\mathcal{L}}}}}}}}}_{3}$ containing both the vertex *u* and the edge *ν*, i.e., *ω* ⊃ *u*, *ν*. Since the lattice ${{{{{{{\mathcal{L}}}}}}}}$ is colorable, the octahedral volume *ω* is antipodally colored, and by definition of the stabilizer and gauge groups, *u* does not belong to *ν*, i.e., $u\cap \nu={{\emptyset}}$. Thus, there is a triangular face *f* of *ω* spanned by *u* and *ν*, i.e., *ω* ⊃ *f* ⊃ *u*, *ν*, and we have ${{{{{{{\mathcal{Q}}}}}}}}(u)\cap {{{{{{{\mathcal{Q}}}}}}}}(\nu )={{{{{{{\mathcal{Q}}}}}}}}(f)$. Since the set ${{{{{{{\mathcal{Q}}}}}}}}(f)$ contains two elements, we conclude that *X*(*u*) and *Z*(*ν*) commute.

Finally, we note that one can show that for any orientable closed 3-manifold the 3D STC has zero logical qubits and that the 3D STC defined on ${{{{{{{{\mathcal{L}}}}}}}}}_{{{{{{{{\rm{cub}}}}}}}}}^{*}$ has one logical qubit. We defer this calculation until Supplementary Note 2. We note that the 3D gauge color code behaves alike, i.e., for any orientable closed 3-manifold it has zero logical qubits.

### Single-shot decoding

In this section, we propose a decoding strategy for the 3D STC, the single-shot MWPM decoder, which consist of two steps: (i) syndrome estimation and (ii) ideal MWPM decoding. We then provide details on our numerical estimates of the performance of the single-shot MWPM decoder. Note that although we focus our discussion on Pauli *X* errors, Pauli *Z* errors can be handled analogously.

#### Single-shot MWPM decoder for the 3D STC

**Fig. 6:**
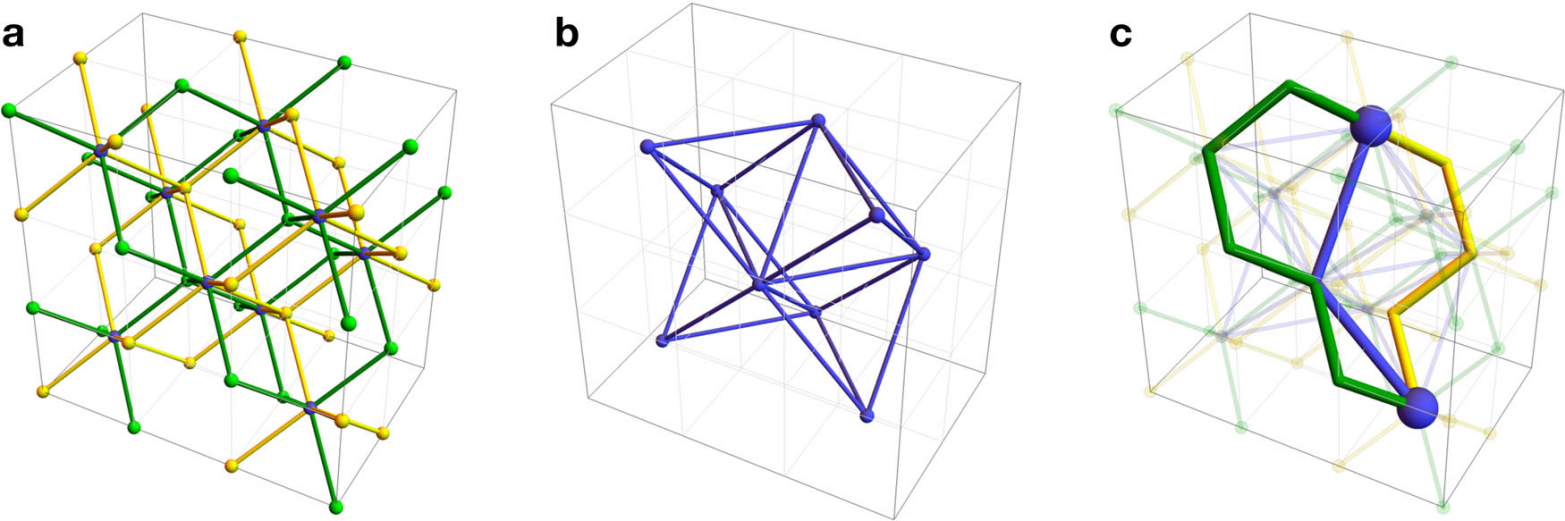
Graphs used in decoding the 3D STC. For the 3D STC defined on a colorable octahedral lattice ${{{{{{{\mathcal{L}}}}}}}}$ we construct **a** the measurement graph *G*_mea_ and **b** the qubit graph *G*_qub_. We use *G*_mea_ and *G*_qub_ in the single-shot MWPM decoder for syndrome estimation and ideal MWPM decoding, respectively. **c** An example of a flux *φ* (green and yellow edges), which may arise for a Pauli *X* error *ϵ* and its stabilizer syndrome ∂_*S*_*ϵ* (blue edges and balls). Note that *φ* satisfies the Gauss law and is not uniquely specified by *ϵ*.

We start this subsection by illustrating the decoding problem for the 3D STC using the measurement and qubit graphs; see Fig. 6. The measurement graph *G*_mea_ = (*V*_mea_, *E*_mea_) is a sublattice of a colorable octahedral lattice ${{{{{{{\mathcal{L}}}}}}}}$, where *V*_mea_ are the *B*, *G* and *Y* vertices of ${{{{{{{\mathcal{L}}}}}}}}$, i.e.,

9 $${V}_{{{{{{{{\rm{mea}}}}}}}}}={{{{{{{{\mathcal{L}}}}}}}}}_{0}^{B}\cup {{{{{{{{\mathcal{L}}}}}}}}}_{0}^{G}\cup {{{{{{{{\mathcal{L}}}}}}}}}_{0}^{Y},$$

and *E*_mea_ is the *BG* and *BY* edges of ${{{{{{{\mathcal{L}}}}}}}}$, i.e.,

10 $${E}_{{{{{{{{\rm{mea}}}}}}}}}={{{{{{{{\mathcal{L}}}}}}}}}_{1}^{BG}\cup {{{{{{{{\mathcal{L}}}}}}}}}_{1}^{BY}.$$

The qubit graph *G*_qub_ = (*V*_qub_, *E*_qub_) is constructed by taking the *B* vertices of the lattice ${{{{{{{\mathcal{L}}}}}}}}$, i.e.,

11 $${V}_{{{{{{{{\rm{qub}}}}}}}}}={{{{{{{{\mathcal{L}}}}}}}}}_{0}^{B},$$

and adding edges between any two different *B* vertices that belong to the same octahedron in ${{{{{{{\mathcal{L}}}}}}}}$. Note that those edges are not present in ${{{{{{{\mathcal{L}}}}}}}}$ however, they are in the one-to-one correspondence with the octahedral volumes of ${{{{{{{\mathcal{L}}}}}}}}$ and thus we can make an identification

12 $${E}_{{{{{{{{\rm{qub}}}}}}}}}={{{{{{{{\mathcal{L}}}}}}}}}_{3}.$$

Let ${V}_{{{{{{{{\rm{mea}}}}}}}}}^{\prime}\subseteq {V}_{{{{{{{{\rm{mea}}}}}}}}}$ and ${V}_{{{{{{{{\rm{qub}}}}}}}}}^{\prime}\subseteq {V}_{{{{{{{{\rm{qub}}}}}}}}}$ denote the sets of all vertices belonging to the boundary $\partial {{{{{{{\mathcal{L}}}}}}}}$ of the lattice ${{{{{{{\mathcal{L}}}}}}}}$. We refer to the vertices in ${V}_{{{{{{{{\rm{mea}}}}}}}}}^{\prime}$ and ${V}_{{{{{{{{\rm{qub}}}}}}}}}^{\prime}$ as the boundary vertices.

It will be convenient to introduce a notion of the *Z*-type gauge flux *φ*, which is defined to be the *Z*-type gauge measurement outcomes in the absence of measurement errors. In Fig. 6c we illustrate a flux *φ*, which may arise from some Pauli *X* error *ϵ*. Local constraints, which we refer to as the Gauss law, arise from the redundancies among gauge generators specified in Fig. 2. We can equivalently interpret this constraint as follows—although the flux *φ* can be random, it forms a collection of strings within *G*_mea_, where each string can only terminate at the boundary vertices. By multiplying all gauge generators supported on edges incident to *v* we obtain the identity operator, and thus the number of operators returning −1 measurement outcome has to be even. From Eq. (8) we conclude that whenever the stabilizer *Z*(*v*) is violated for some vertex $v\in {V}_{{{{{{{{\rm{qub}}}}}}}}}\setminus {V}_{{{{{{{{\rm{qub}}}}}}}}}^{\prime}$, then the number of gauge generators supported on *BG* edges (or *BY* edges) incident to *v* and returning −1 measurement outcomes, has to be odd. Thus, for the given Pauli *X* error *ϵ* and the flux *φ* the corresponding stabilizer syndrome *σ* can be either found as the endpoints of strings in *ϵ* or, equivalently, as the vertices incident to an odd number of *BG* edges (or *BY* edges) in *φ*.

We remark that the physics of the gauge flux is qualitatively similar in the 3D STC and the 3D gauge color code^20,39,96,97^. However, the behavior of the 3D STC gauge flux is substantially simpler. For instance, in the 3D STC, the *Z*-type gauge flux has two types (the flux type is given by the color of the corresponding edge in the measurement graph *G*_mea_), compared to six types for the 3D gauge color code. Also, the flux in the 3D gauge color code may have branching points where fluxes of three certain types meet; this phenomenon is not present in the 3D STC.

We formalize the above discussions as follows. Let *C*_*G*_, *C*_*Q*_, *C*_*M*_, *C*_*S*_*,* and *C*_*R*_ be ${{\mathbb{F}}}_{2}$-vector spaces corresponding, respectively, to the sets of *X*-type gauge operators, qubits, measured *Z*-type gauge operators, independent *Z*-type stabilizer generators, and independent relations between measured *Z*-type gauge operators. The relations correspond to the local Gauss law constraints described above. We refer to the elements of the vector spaces *C*_*G*_, *C*_*Q*_ ⊕ *C*_*M*_, *C*_*M*_*,* and *C*_*S*_ ⊕ *C*_*R*_ as gauge operators, errors, measurement outcomes (the measurement outcome is defined as the set of all measured *Z*-type gauge operators returning a −1 outcome), and syndromes, respectively. Every error *ϵ* ⊕ *μ* ∈ *C*_*Q*_ ⊕ *C*_*M*_ consists of the Pauli *X* error *ϵ* and the measurement error *μ*. Similarly, every syndrome *σ* ⊕ *ω* ∈ *C*_*S*_ ⊕ *C*_*R*_ consists of the stabilizer syndrome *σ*, which is the set of violated stabilizers, and the relation syndrome *ω*, which is the set of violated relations. We choose the linear maps in Eq. (13) in such a way that: (i) the support of any *X*-type gauge operator *γ* ∈ *C*_*G*_ is ∂_*Q*_*γ* ∈ *C*_*Q*_, (ii) the set of *Z*-type gauge operators anticommuting with any Pauli *X* error *ϵ* ∈ *C*_*Q*_ is *δ*_*M*_*ϵ* ∈ *C*_*M*_, (iii) the stabilizer syndrome and the relation syndrome corresponding to the measurement outcome *ζ* ∈ *C*_*M*_ are *δ*_*S*_*ζ* ∈ *C*_*S*_ and *δ*_*R*_*ζ* ∈ *C*_*R*_.13We define ∂_*S*_ = *δ*_*S*_*δ*_*M*_ and require that

14 $${\partial }_{S}{\partial }_{Q}=0,$$

as the stabilizer syndrome of any *X*-type gauge operator has to be trivial. We also require that

15 $${\delta }_{R}{\delta }_{M}=0,$$

as, by definition, the relation syndrome of any Pauli *X* error is trivial.

Now, we are ready to introduce the single-shot MWPM decoder for the 3D STC defined on the lattice ${{{{{{{\mathcal{L}}}}}}}}$. It consists of the following two steps.

(i) *Syndrome estimation*: We exploit the consistency checks on the measurement outcomes of *Z*-type gauge operators. Namely, for the given measurement outcome *ζ* we find the minimum-weight estimate $\widehat{\mu }$ of the measurement error *μ*, i.e.,
  16 $${\widehat{\mu}}=\mathop{{{{{{\mathrm{arg}}}}}}\, {{{{{\mathrm{min}}}}}}}\limits_{\mu ^{\prime} \in {C}_{M}:{\partial }_{R}\mu ^{\prime}={\partial }_{R}\zeta }|\mu ^{\prime}|.$$
  Finding $\widehat{\mu }$ is an instance of the MWPM problem for the measurement graph *G*_mea_. Then, we compute an estimate $\widehat{\sigma }$ of the stabilizer syndrome ∂_*S*_*ϵ* of the error *ϵ* as follows:
  17 $$\widehat{\sigma }={\delta }_{S}(\zeta+\widehat{\mu }).$$
(ii) *Ideal MWPM decoding*: For the given syndrome estimate $\widehat{\sigma }$ we find the corresponding minimum-weight recovery operator *χ*, i.e.,
  18 $$\chi=\mathop{{{{{{\mathrm{arg}}}}}}\, {{{{{\mathrm{min}}}}}}}\limits_{\chi ^{\prime} \in {C}_{Q}:{\partial }_{S}\chi ^{\prime}=\widehat{\sigma }}|\chi ^{\prime}|.$$
  Finding *χ* is an instance of the MWPM problem for the qubit graph *G*_qub_.

**Fig. 7:**
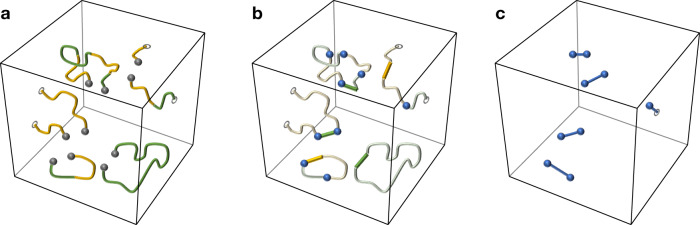
An illustration of the single-shot MWPM decoder. **a** Due to the measurement error *μ*, the measurement outcome *ζ* (yellow and green lines) has non-zero relation syndrome *δ*_*R*_*ζ* (gray dots), i.e., *ζ* violates the Gauss law. **b** In step (i), we first find the minimum-weight estimate $\widehat{\mu }$ (yellow and green lines) of *μ*, such that $\zeta+\widehat{\mu }$ has trivial relation syndrome, i.e., ${\delta }_{R}(\zeta+\widehat{\mu })=0$. Then, we compute a syndrome estimate $\widehat{\sigma }$ (blue dots). **c** In step (ii), we find the minimum-weight recovery operator *χ* (blue lines) for $\widehat{\sigma }$.

We illustrate how the single-shot MWPM decoder works in Fig. 7a–c. We emphasize that in the presence of measurement errors it is likely that there will be some residual Pauli *X* error left in the system after applying the single-shot MWPM decoder.

#### Numerical simulations of the performance

We numerically estimate the performance of the single-shot MWPM decoder for the 3D STC on the lattice ${{{{{{{{\mathcal{L}}}}}}}}}_{{{{{{{{\rm{cub}}}}}}}}}^{*}$ from Sec. II, which is based on the cubic lattice with open boundary conditions. We assume the independent and identically distributed bit-flip noise with probability *p* and set the measurement error rate to match the bit-flip error rate, i.e., *q* = *p*.

**Fig. 8:**
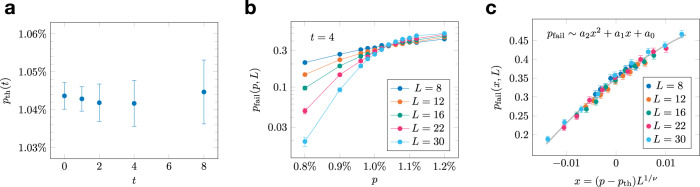
Numerical estimates of the single-shot MWPM decoder threshold for the 3D STC on ${{{{{{{{\mathcal{L}}}}}}}}}_{{{{{{{{\rm{cub}}}}}}}}}^{*}$. **a** The threshold *p*_th_(*t*) does not change noticeably with the number of correction cycles *t*. We estimate the storage threshold *p*_STC_ ≈ 1.045%. **b** The failure probability *p*_fail_(*p*, *D*) after *t* = 4 correction cycles, where *p* is the bit-flip error rate, *D* is the code distance, and we set the measurement error rate *q* = *p*. In **c**, we show the same data using the rescaled variable *x* = (*p*−*p*_th_(*t*))*D*^1/*μ*^, where the fitting parameters are *μ* = 1.2(1) and *p*_th_(*t*) = 0.01042(6), giving the *t* = 4 data point in (**a**). In each plot, the error bars show standard error estimates.

In our Monte Carlo simulations, we first perform a fixed number of correction cycles *t*. We start by initializing the residual error to zero, i.e., *ρ* = 0. Then, in each correction cycle we: (i) update the existing residual error *ρ* by adding a randomly chosen (according to the error model described above) error *ϵ* to it, (ii) select a gauge operator *γ* uniformly at random, (iii) choose a random measurement error *μ*, (iv) find the measurement outcome *ζ* = *δ*_*M*_*ρ* + *μ* + *δ*_*M*_∂_*Q*_*γ*, (v) use the single-shot MWPM decoder to find a recovery operator *χ*, (vi) update the residual error *ρ* by adding the recovery operator *χ* to it. After *t* error correction cycles, we add a randomly chosen error to the residual error, extract the measurement outcome with no measurement error, use the single-shot MWPM decoder to find a recovery operator, which returns the state to the code space, and, finally, check for a logical error. This, in turn, allows us to estimate the threshold *p*_th_(*t*) (see Fig. 8b, c). Note that *p*_th_(0) corresponds to the code capacity threshold. Moreover, we are interested in the storage threshold of the 3D STC, which we define as the limit of the threshold *p*_th_(*t*) as the number of correction cycles *t* goes to infinity, i.e.,

19 $${p}_{{{{{{{{\rm{STC}}}}}}}}}=\mathop{\lim }\limits_{t\to \infty }{p}_{{{{{{{{\rm{th}}}}}}}}}(t).$$

We observe that the threshold *p*_th_(*t*) does not change noticeably with *t* (see Fig. 8a). We estimate the storage threshold to be *p*_STC_ ≈ 1.045%, and we note that this is approximately five times the lower bound on the storage threshold (for *t* = 0 cycles) derived in Supplementary Lemma 3.

We also investigate the behavior of the single-shot MWPM decoder in the subthreshold regime. We use the following ansatz:

20 $${p}_{{{{{{{{\rm{fail}}}}}}}}}(p,D,t)=(t+1){\left(\frac{p}{{p}_{{{{{{{{\rm{th}}}}}}}}}(t)}\right)}^{\alpha (t){D}^{\beta (t)}}$$

for the logical failure probability *p*_fail_(*p*, *D*, *t*) as a function of the error rate *p*, the code distance *D*, and the number of correction cycles *t*. This ansatz is derived from assuming that the logical failure probability in each cycle is ${p}_{{{{{{{{\rm{fail}}}}}}}}}={(p/{p}_{{{{{{{{\rm{th}}}}}}}}})}^{\alpha {D}^{\beta }}$. For *t* cycles, a first-order expansion (assuming small *p*_fail_) gives Eq. (20), where we have added one to the number of cycles to account for logical failures during readout.

**Fig. 9:**
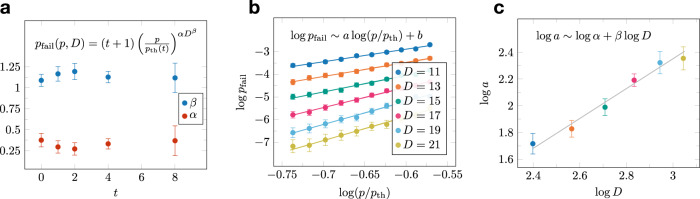
Subthreshold behavior of the single-shot MWPM decoder. **a** We estimate the parameters *α*(*t*) and *β*(*t*) of the ansatz in Eq. (20) for the logical failure probability. We find that both *α*(*t*) and *β*(*t*) are stable across the range of simulated values of *t*. **b**, **c** An illustration of the fitting procedure for *t* = 8. **b** We plot $\log {p}_{{{{{{{{\rm{fail}}}}}}}}}$ as a function of $\log p/{p}_{{{{{{{{\rm{th}}}}}}}}}$ for different values of the code distance *D*. We fit the data to straight lines to obtain estimates of *a* as a function of *D*. **c** We plot $\log a=\log \alpha (t)+\beta (t)\log D$ as a function of $\log D$ and fit to a straight line to estimate *α*(*t*) and *β*(*t*). In each plot, the error bars show standard error estimates.

In Eq. (20), *α*(*t*) and *β*(*t*) are fitting parameters, which may depend on *t*, and *p*_th_(*t*) is obtained from threshold fits similar to the ones depicted in Fig. 8b, c. To estimate *α*(*t*) and *β*(*t*), we first fix *t* and take the logarithm of both sides of Eq. (20) to obtain

21 $$\log {p}_{{{{{{{{\rm{fail}}}}}}}}}(p,D,t)=\log (t+1)+a\log (p/{p}_{{{{{{{{\rm{th}}}}}}}}}(t)),$$

where we introduce *a* = *α*(*t*)*D*^*β*(*t*)^ Then, for different values of *D*, we plot $\log {p}_{{{{{{{{\rm{fail}}}}}}}}}(p,D,t)$ as a function of $\log (p/{p}_{{{{{{{{\rm{th}}}}}}}}}(t))$ and fit to a straight line to estimate *a* (see Fig. 9b). We then take the logarithm again and obtain

22 $$\log a=\log \alpha (t)+\beta (t)\log D.$$

Finally, we plot $\log a$ as a function of $\log D$ and fit to a straight line to get *α*(*t*) and *β*(*t*) (see Fig. 9c). As the weight of the smallest logical operator is equal to *D*, we expect *β*(*t*) ~ 1, which is indeed what we observe. Furthermore, the values of *α*(*t*) and *β*(*t*) are stable for the values of *t* that we simulated (see Fig. 9a).

### Proof of single-shot QEC

In this section, we give an informal statement of our theorem proving that single-shot QEC is possible with the 3D STC, and we discuss its interpretation.

We begin with some definitions. Let *p*: 2^*A*^ → [0, 1] be a discrete probability distribution. We say that *p* is *τ*-bounded iff for any set *B* ⊆ *A* the probability that a set $B^{\prime} \subseteq A$ drawn according to *p* contains *B* is at most *τ*^∣*B*∣^, i.e.,

23 $$\forall B\subseteq A:\mathop{\sum}\limits_{B^{\prime} \supseteq B}p(B^{\prime} )\le {\tau }^{|B|}.$$

We can describe any stochastic Pauli *X* noise model ${{{{{{{\mathcal{N}}}}}}}}$ as a Pauli *X* channel admitting a Kraus representation with an associated probability distribution ${p}_{{{{{{{{\mathcal{N}}}}}}}}}$. We say that ${{{{{{{\mathcal{N}}}}}}}}$ is *τ*-bounded iff the corresponding probability distribution ${p}_{{{{{{{{\mathcal{N}}}}}}}}}$ is. For example, the bit-flip noise with probability *τ* is *τ*-bounded, as in this case, we have equality in Eq. (23).

We describe ideal error correction by the channel ${{{{{{{{\mathcal{R}}}}}}}}}_{0}$, which consists of a projector onto the subspace with a stabilizer syndrome *σ* followed by the application of the recovery operator *R*_*σ*_ found by the MWPM decoder. This allows us to define the failure probability for a Pauli *X* channel ${{{{{{{\mathcal{N}}}}}}}}$ as the probability that the channel ${{{{{{{{\mathcal{R}}}}}}}}}_{0}\circ {{{{{{{\mathcal{N}}}}}}}}\circ {{{\Pi }}}_{0}^{{{{{{{{\mathcal{S}}}}}}}}}$ implements a non-trivial logical Pauli operator, where ${{{\Pi }}}_{0}^{{{{{{{{\mathcal{S}}}}}}}}}$ is the codespace projector. For sufficiently small *τ*, the failure probability of the bit-flip noise scales as *τ*^⌊(*d*+1)/2⌋^, where *d* is the code distance. We can now define a class of channels ${{\mathbb{N}}}_{\tau,\epsilon }$ comprising all Pauli *X* channels whose failure probability is upper-bounded by *ϵ* and whose associated probability distributions ${p}_{{{{{{{{\mathcal{N}}}}}}}}}$ are (up to a technical equivalence condition) is *τ*-bounded.

Finally, we use ${{{{{{{\mathcal{R}}}}}}}}$ to denote a single-shot MWPM decoding channel whose measurement errors have an associated probability distribution ${p}_{{{{{{{{\mathcal{R}}}}}}}}}$. This allows us to define ${{\mathbb{R}}}_{\eta }$ to be the set of all MWPM decoding channels ${{{{{{{\mathcal{R}}}}}}}}$ whose corresponding probability distributions ${p}_{{{{{{{{\mathcal{R}}}}}}}}}$ are *η*-bounded.

#### Theorem 1

(Informal version). *For sufficiently small* *η* *and* *τ* *there exist* $\tau ^{\prime}$ *and* $\epsilon ^{\prime}$ *satisfying*

24 $$\mathop{\lim }\limits_{\eta \to 0}\tau ^{\prime}=0,\quad \mathop{\lim }\limits_{L\to \infty }\epsilon ^{\prime}=\epsilon,$$

*such that the following inclusion holds*

25 $${{\mathbb{R}}}_{\eta }\circ {{\mathbb{N}}}_{\tau,\epsilon }\circ {{{\Pi }}}_{0}^{{{{{{{{\mathcal{S}}}}}}}}}\subseteq {{\mathbb{N}}}_{\tau ^{\prime},\epsilon ^{\prime} }\circ {{{\Pi }}}_{0}^{{{{{{{{\mathcal{S}}}}}}}}}.$$

We defer the proof until Supplementary Note 4. Crucially, Theorem 1 says that the parameter $\tau ^{\prime}$ governing the residual noise strength can be made arbitrarily small just by reducing the measurement error parameter *η* of the single-shot MWPM decoder. Moreover, the failure probability $\epsilon ^{\prime}$ for the residual noise can be made arbitrarily close to the failure probability for the original noise *ϵ* just by increasing the linear size *L* of the system. Furthermore, we can use Theorem 1 to show that after *n* rounds of Pauli *X* noise and single-shot MWPM decoding we have

26 $${({{\mathbb{R}}}_{\eta }\circ {{\mathbb{N}}}_{\tau,\epsilon })}^{n}\circ {{{\Pi }}}_{0}^{{{{{{{{\mathcal{S}}}}}}}}}\subseteq {{\mathbb{N}}}_{\tau ^{\prime},\epsilon (n)}\circ {{{\Pi }}}_{0}^{{{{{{{{\mathcal{S}}}}}}}}},$$

where *ϵ*(*n*) can be made arbitrarily close to *nϵ* by increasing *L* and again $\mathop{\lim }\nolimits_{\eta \to 0}\tau ^{\prime}=0$. In the case of bit-flip noise, for sufficiently small *τ* we can achieve a failure probability *ϵ*(*n*) → *nτ*^⌊(*d*+1)/2⌋^, which scales as the failure probability of applying the noise *n* times. We note that the condition on $\tau ^{\prime}$ is also important, as we need $\tau ^{\prime}$ to stay below the error threshold of the code subject to bit-flip noise. Equation (26) rigorously establishes that the residual noise in the 3D STC does not accumulate in an uncontrollable way after multiple rounds of single-shot QEC, and therefore the logical information encoded in the 3D STC will be protected for a long time.

## Supplementary information

Supplementary Information

## Data Availability

The data generated in this study have been deposited in the Zenodo database 10.5281/zenodo.7087715.

## Code availability

The code that supports the findings of this study is available from the corresponding author upon request.
