# Supplementary material for: Single-shot quantum error correction with the three-dimensional subsystem toric code
Source: Nat Commun. 2022 Oct 21;13:6272. doi: 10.1038/s41467-022-33923-4 (PMC9586949; doi:10.1038/s41467-022-33923-4)
Supplement: Supplementary file 1 — Supplementary Information [file 41467_2022_33923_MOESM1_ESM.pdf]

Supplementary Information for "Single-shot quantum error correction with the three-dimensional subsystem toric code"

Aleksander Kubica[1,2,3,4] and Michael Vasmer[1,2]
[1] Perimeter Institute for Theoretical Physics, Waterloo, ON N2L 2Y5, Canada
[2] Institute for Quantum Computing, University of Waterloo, Waterloo, ON N2L 3G1, Canada
[3] AWS Center for Quantum Computing, Pasadena, CA 91125, USA
[4] California Institute of Technology, Pasadena, CA 91125, USA

SUPPLEMENTARY NOTE 1: REDUCING THE WEIGHT OF GAUGE OPERATORS

The realization of the 3D STC on the lattice $\mathcal{L}^*_{\text{cub}}$ is very simple, however the gauge group $\mathcal{G}$ of the 3D STC cannot be generated by gauge operators of weight at most three. Namely, along the top and bottom boundaries of $\mathcal{L}^*_{\text{cub}}$ there are gauge operators of weight four; see Fig. 1(c). Ideally, we would like all gauge operators to be of weight at most three, as in such a case the noisy measurement circuits would not spread errors [1]. We reduce the weight of the gauge operators by using an idea from Ref. [2], where a version of the 2D toric code with only weight-three operators was proposed.

To reduce the weight of gauge operators at the top and bottom boundaries of the lattice $\mathcal{L}^*_{\text{cub}}$, we first place some unentangled ancilla qubits $\mathcal{A}_S \cup \mathcal{A}_G$ on those boundaries; see Supplementary Figure 1(a). Every ancilla qubit $i \in \mathcal{A}_S$ is prepared in the state $|0\rangle$ and thus is stabilized by a single-qubit Pauli Z operator; every other ancilla qubit $j \in \mathcal{A}_G$ is treated as a gauge qubit. Hence, we arrive at an augmented subsystem code with the following gauge group

$$\mathcal{G}_{\text{aug}} = \langle G, Z_i, X_j, Z_j \mid G \in \mathcal{G}, i \in \mathcal{A}_S, j \in \mathcal{A}_G \rangle. \quad (1)$$

Then, we implement a unitary U via a constant-depth circuit composed of four rounds of geometrically-local CNOT gates. One can straightforwardly verify that the generators of the gauge group $U\mathcal{G}_{\text{aug}}U^\dagger$ can be chosen in such a way that each of them has weight at most three. In Supplementary Figure 1(b) we depict the choice of generators fully supported within the top and bottom boundaries. We remark that the subsystem code $U\mathcal{G}_{\text{aug}}U^\dagger$ and the 3D STC on $\mathcal{L}^*_{\text{cub}}$ from the previous subsection are equivalent in the sense of a local unitary transformation and adding or removing ancilla qubits [2–5].

SUPPLEMENTARY NOTE 2: CALCULATING THE NUMBER OF LOGICAL QUBITS ENCODED IN THE 3D STC

In this section we show that the 3D STC defined on a tessellation of the 3-sphere has zero logical qubits and that the 3D STC defined on $\mathcal{L}^*_{\text{cub}}$ has one logical qubit.

The following lemma proves useful in calculating the number of constituents of a colorable octahedral lattice.

Lemma 1. *Let $\mathcal{L}$ be a colorable octahedral lattice without boundary. Then, the following conditions on the number of constituents of $\mathcal{L}$ hold*

(i) $|\mathcal{L}_2| = 4|\mathcal{L}_3|$,

(ii) $|\mathcal{L}_1| = |\mathcal{L}_1^{RG}| + |\mathcal{L}_1^{RY}| + |\mathcal{L}_1^{BG}| + |\mathcal{L}_1^{BY}| + |\mathcal{L}_1^{RB}|$,

(iii) $2|\mathcal{L}_0^G| + 2|\mathcal{L}_0^Y| - |\mathcal{L}_1| + |\mathcal{L}_1^{RB}| + 2|\mathcal{L}_3| = 0$.

Proof. To show (i), we observe that each face of $\mathcal{L}$ is shared between two octahedral volumes and every octahedral volume has eight faces. To show (ii), we observe that there are no GY edges in $\mathcal{L}$, as every octahedral volume in $\mathcal{L}$ is antipodally colored. To show (iii), we construct a new tessellation of the manifold corresponding to $\mathcal{L}$. We first split every octahedral volume $\omega \in \mathcal{L}_3$ into two pyramids by inserting into ω a face $f(\omega)$ glued along the cycle consisting of four RB edges. Then, for every G or Y vertex $v \in \mathcal{L}_0^G \cup \mathcal{L}_0^Y$ we find all the pyramids containing v and merge them into a single three-dimensional cell $c(v)$, whose boundary corresponds to the collection of the bases of these pyramids. Note that this step removes all the edges and vertices of color different than RB and R or B, respectively. The resulting tessellation $\widehat{\mathcal{L}}$ consists of the vertices $\mathcal{L}_0^R \cup \mathcal{L}_0^B$, edges $\mathcal{L}_1^{RB}$, faces $\{f(\omega) | \omega \in \mathcal{L}_3\}$, and volumes $\{c(v) | v \in \mathcal{L}_0^G \cup \mathcal{L}_0^Y\}$. Since for $\mathcal{L}$ and $\widehat{\mathcal{L}}$ the Euler characteristic is the same (and equal to zero), we obtain

$$\begin{aligned} &|\mathcal{L}_0| - |\mathcal{L}_1| + |\mathcal{L}_2| - |\mathcal{L}_3| \\ &\quad = |\mathcal{L}_0^R| + |\mathcal{L}_0^B| - |\mathcal{L}_1^{RB}| + |\mathcal{L}_3| - |\mathcal{L}_0^G| - |\mathcal{L}_0^Y|, \end{aligned} \quad (2)$$

and by using (i) and rearranging the terms we finally obtain (iii). □

Now, for simplicity, we assume that the lattice $\mathcal{L}$ is obtained by tessellating a 3-sphere. We can verify that the 3D STC defined on $\mathcal{L}$ has no logical qubits. Since stabilizer generators correspond to R and B vertices of $\mathcal{L}$ and there are two relations between them, namely

$$\prod_{u \in \mathcal{L}_0^R} X(u) = \prod_{v \in \mathcal{L}_0^R} Z(v) = I, \quad (3)$$

thus the number of independent generators of the stabilizer group $\mathcal{S}$ is

$$\log_2 |\mathcal{S}| = |\mathcal{L}_0^R| + |\mathcal{L}_0^B| - 2. \quad (4)$$

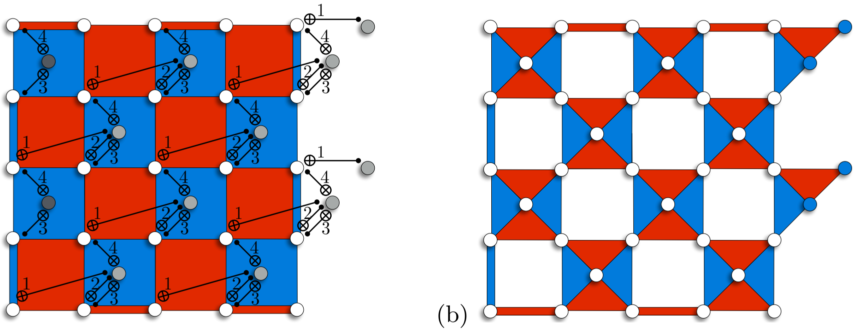

Supplementary Figure 1. (a) To reduce the weight of weight-four gauge operators along the top and bottom boundaries of the lattice $\mathcal{L}^*_{\mathrm{cub}}$ we introduce ancilla qubits $\mathcal{A}_S$, which are in the state $|0\rangle$ (dark gray dots), as well as gauge qubits $\mathcal{A}_G$ (light gray dots). We then implement a unitary U via a circuit comprising four rounds of CNOT gates. The circuit has depth three as CNOT gates in rounds two and three can be combined. (b) The choice of gauge generators of the subsystem code $U\mathcal{G}_{\mathrm{aug}}U^\dagger$ that are fully supported within the top and bottom boundaries. Some qubits along the right side (blue dots) support single-qubit Pauli Z operators.

We also find the following four types of relations for gauge generators

$$\forall u \in \mathcal{L}_0^R: \quad \prod_{\mu \in \mathcal{L}_1^{RG} \cup \mathcal{L}_1^{RY} : \mu \supset u} X(\mu) = I, \tag{5}$$

$$\forall v \in \mathcal{L}_0^B: \quad \prod_{\mu \in \mathcal{L}_1^{BG} \cup \mathcal{L}_1^{BY} : \mu \supset v} Z(\mu) = I, \tag{6}$$

$$\forall w \in \mathcal{L}_0^G: \quad \prod_{\mu \in \mathcal{L}_1^{RG} : \mu \supset w} X(\mu) = \prod_{\mu \in \mathcal{L}_1^{BG} : \mu \supset w} Z(\mu) = I, \tag{7}$$

$$\forall w \in \mathcal{L}_0^Y: \quad \prod_{\mu \in \mathcal{L}_1^{RY} : \mu \supset w} X(\mu) = \prod_{\mu \in \mathcal{L}_1^{BY} : \mu \supset w} Z(\mu) = I. \tag{8}$$

However, the above relations are not all independent; rather, we overcount them by 2. Since gauge generators correspond to RG, RY, BG and BY edges of $\mathcal{L}$, thus the number of independent generators of the gauge group $\mathcal{G}$ is

$$\begin{aligned} \log_2 |\mathcal{G}| =& |\mathcal{L}_1^{RG}| + |\mathcal{L}_1^{RY}| + |\mathcal{L}_1^{BG}| + |\mathcal{L}_1^{BY}| \\ & - |\mathcal{L}_0^R| - |\mathcal{L}_0^B| - 2|\mathcal{L}_0^G| - 2|\mathcal{L}_0^Y| + 2 \\ =& 2|\mathcal{L}_3| - |\mathcal{L}_0^R| - |\mathcal{L}_0^B| + 2, \end{aligned} \tag{9}$$

where we use Lemma 1. We finally obtain that the number of logical qubits encoded into the 3D STC on the lattice $\mathcal{L}$ is

$$K = N - \frac{1}{2}(\log_2 |\mathcal{G}| + \log_2 |\mathcal{S}|) = 0. \tag{10}$$

We remark that we could also consider the 3D STC on a colorable octahedral lattice, which is a tessellation of any orientable closed 3-manifold, not necessarily a 3-sphere. In such a case, however, the stabilizer group would not only be generated by geometrically-local generators in Eq. (6), but also by non-local ones corresponding to non-trivial elements of the second homology group of the manifold. At the same time, for each non-trivial element of the second homology group we would find an independent relation for gauge generators. Thus, we would conclude that the 3D STC has zero logical qubits. This is why in the Results section we consider the 3D STC defined on the lattice $\mathcal{L}^*_{\mathrm{cub}}$ with open boundary conditions instead of a simpler translationally-invariant lattice, such as the cubic lattice with periodic boundary conditions.

We have just seen that the 3D STC defined on a colorable octahedral lattice $\mathcal{L}$, which is a tessellation of a 3-sphere, has no logical qubits. To obtain the 3D STC with one logical qubit, we first construct a new lattice $\mathcal{L}'$ by removing one octahedral volume from $\mathcal{L}$. This procedure is analogous to the construction of the color code with one logical qubit [6]. Note that the lattice $\mathcal{L}'$ is a tessellation of the three-dimensional ball, and its boundary $\partial\mathcal{L}'$ corresponds to the boundary of the removed octahedral volume. The gauge group $\mathcal{G}'$ is generated by X- and Z-type operators supported on RG or RY and BG or BY edges in the interior of $\mathcal{L}'$, namely

$$\begin{aligned} \mathcal{G}' = \langle X(\mu), Z(\nu) \,|\, & \mu \in \mathcal{L'}_1^{RG} \cup \mathcal{L'}_1^{RY} \setminus \partial\mathcal{L'}_1, \\ & \nu \in \mathcal{L'}_1^{BG} \cup \mathcal{L'}_1^{BY} \setminus \partial\mathcal{L'}_1 \rangle. \end{aligned} \tag{11}$$

One can verify that the stabilizer group is generated by X- and Z-type operators supported on R and B vertices in the interior of $\mathcal{L}'$, namely

$$\mathcal{S}' = \langle X(u), Z(v) \mid u \in \mathcal{L'}_0^R \setminus \partial\mathcal{L'}_0, v \in \mathcal{L'}_0^B \setminus \partial\mathcal{L'}_0 \rangle. \tag{12}$$

Compared to the 3D STC defined on $\mathcal{L}$, the number of physical qubits is reduced by one. Also, we discard four stabilizer generators supported on vertices of the boundary $\partial\mathcal{L}$. Since the stabilizer generators no longer satisfy the relations in Eq. (3), we thus obtain that the number of independent stabilizer generators is reduced by two. Similarly, we discard eight gauge generators supported on edges belonging to $\partial\mathcal{L}$ and the remaining gauge generators no longer satisfy eight relations out of the ones in Eqs. (5)-(8). The remaining relations are, however, independent, and thus the number of independent gauge generators is reduced by two. Combining the above and

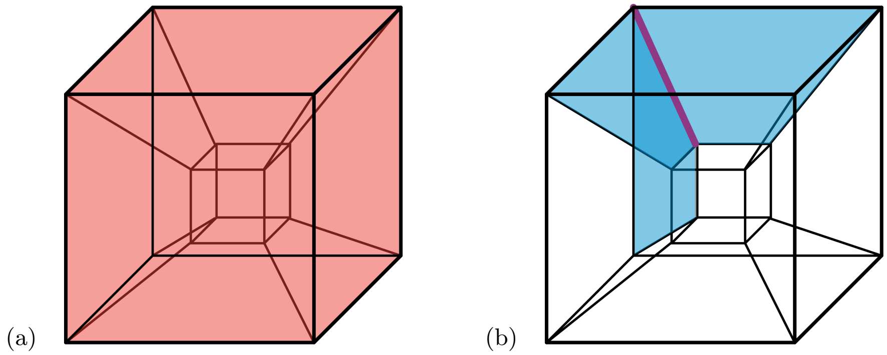

Supplementary Figure 2. Stabilizers and gauge operators of the 4D STC. The support of (a) an X-type stabilizer and (b) Z-type gauge operator in the 4D STC with qubits placed on faces. The X-type stabilizer acts on the 24 qubits contained in a red 4-hypercube. The Z-type gauge operator associated with a blue 4-hypercube and an edge contained in this hypercube (purple) acts on the three qubits (shaded in blue) that are contained in the hypercube and also contain the edge.

using Eq. (10), we obtain that the 3D STC defined on the lattice $\mathcal{L}'$ with boundary has one logical qubit. We then immediately conclude that the 3D STC defined on the lattice $\mathcal{L}^*_{\text{cub}}$ from the Results section also has one logical qubit, as it is an example of this construction (except in the dual lattice). Lastly, we remark that by removing more octahedral volumes we could encode more logical qubits.

SUPPLEMENTARY NOTE 3: STC IN HIGHER DIMENSIONS

In this appendix, we present a generalization of the STC to $d \geq 3$ dimensions. We start by returning to and formalizing the picture of the 3D STC that we used in the Results section. Next, we discuss the 4D STC and show that it is related to the 4D stabilizer toric codes with a transversal four-qubit control-Z gate described in Ref. [7]. Finally, we sketch a proof that the STC defined on a d-dimensional hypercubic lattice with periodic boundary conditions has zero logical qubits.

Recasting the STC

Let $\mathcal{L}$ denote a d-dimensional hypercubic lattice with periodic boundary conditions and linear length L, where L is even. As in the Methods section we use $\mathcal{L}_i$ to denote the i-hypercubes of $\mathcal{L}$. We can color the d-hypercubes of $\mathcal{L}$ in red and blue such that no two d-hypercubes sharing a $(d-1)$-hypercube have the same color. We denote the corresponding sets of d-hypercubes as $\mathcal{L}_d^R$ and $\mathcal{L}_d^B$, respectively. We place qubits on the $(d-2)$-hypercubes of $\mathcal{L}$. Anticipating the definition of the STC, for any i-hypercube $\mu \in \mathcal{L}_i$, we define $\mathcal{Q}(\mu)$ to be the set of qubits on all the $(d-2)$-hypercubes that either contain μ if $i < d-2$ or are contained in μ if $i \geq d-2$. Namely,

$$\mathcal{Q}(\mu) = \begin{cases} \{\omega \in \mathcal{L}_{d-2} | \omega \supset \mu\} & \text{if } i < d-2, \\ \{\omega \in \mathcal{L}_{d-2} | \omega \subseteq \mu\} & \text{otherwise.} \end{cases} \tag{13}$$

As in the Methods section, when we say that an operator is associated with δ we mean that it is supported on the set of qubits $\mathcal{Q}(\delta)$ and write $X(\delta) = \prod_{\omega \in \mathcal{Q}(\delta)} X_\omega$, where X_ω denotes a Pauli X operator acting on the qubit on the $(d-2)$-hypercube ω. In the next section, we abuse the notation somewhat and talk about operators being supported on i-cells of various lattices, but it should be clear from the context what we mean.

The gauge group of the d-dimensional STC is generated by operators associated with pairs comprising d-hypercubes and $(d-3)$-hypercubes. Namely, for each d-hypercube $\mu \in \mathcal{L}_d^R$ and $(d-3)$-hypercube $\lambda \subset \mu$, we have the gauge operator

$$X(\mu, \lambda) = \prod_{\omega \in \mathcal{Q}(\mu) \cap \mathcal{Q}(\lambda)} X_\omega. \tag{14}$$

Likewise for each d-hypercube $\nu \in \mathcal{L}_d^B$ and $(d-3)$-hypercube $\kappa \subset \nu$, we have the gauge operator

$$Z(\nu, \kappa) = \prod_{\omega \in \mathcal{Q}(\nu) \cap \mathcal{Q}(\kappa)} Z_\omega. \tag{15}$$

Thus, the gauge group of the d-dimensional STC is

$$\begin{aligned} \mathcal{G} = \langle X(\mu, \lambda), Z(\nu, \kappa) \, | \mathcal{L}_d^R \ni \mu \supset \lambda \in \mathcal{L}_{d-3}, \\ \mathcal{L}_d^B \ni \nu \supset \kappa \in \mathcal{L}_{d-3} \rangle. \end{aligned} \tag{16}$$

Since the number of i-hypercubes contained in a d-hypercube is $2^{d-i}\binom{d}{d-i}$, there are $2^3\binom{d}{3}|\mathcal{L}_d|$ gauge generators of $\mathcal{G}$. Note that not all of them are independent. Also, one can check that the above definitions are equivalent to the pictorial representation shown in Figs. 2a and 2b for $d = 3$. We illustrate the construction in 4D in Supplementary Figure 2.

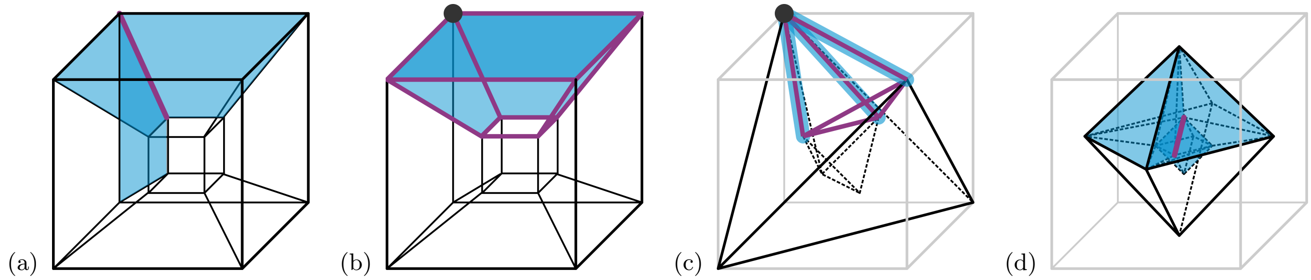

Supplementary Figure 3. The evolution of Z-type gauge generator through three lattice transformations. (a) A 4-hypercube of $\mathcal{L}$, the original 4D STC lattice, with qubits on faces. We illustrate the support (shaded blue) of the gauge operator associated with the 4-hypercube and an edge contained in this hypercube (purple). (b) A 4-hypercube of $\mathcal{L}^*$, the dual of $\mathcal{L}$ (qubits are still on faces). The gauge operator is now associated with a vertex (dark gray circle) and a 3-hypercube containing this vertex (outlined in purple). The faces in its support are shaded in blue. (c) A 4-cell of $\mathcal{L}^*_{\rm alt}$, the alternation of $\mathcal{L}^*$, with qubits on edges. We show the outer 3-cell of (b) as a guide to the eye. Here, the gauge operator is associated with a vertex (dark gray circle) and a 3-cell containing this vertex (outlined in purple). The edges in its support are highlighted in blue. (d) A 4-cell of $\mathcal{L}_{\rm opx}$, the dual of $\mathcal{L}^*_{\rm alt}$, with qubits on 3-cells. Here, the gauge operator is associated with the 4-cell and an edge contained in this 4-cell (purple). The 3-cells in its support are shaded in blue. To avoid clutter, in (c) and (d) we draw only a subset of the edges of the 4-cells.

The stabilizer group of the d-dimensional STC is generated by operators associated with d-hypercubes and $(d-1)$-dimensional hyperplanes, namely

$$\mathcal{S} = \langle X(\mu), Z(\nu), X(\pi_i), Z(\pi_i) \mid \mu \in \mathcal{L}^R_d, \nu \in \mathcal{L}^B_d, i \in \{1,\ldots,d\}\rangle, \tag{17}$$

where $X(\pi_i)$ denotes a product of Pauli X operators acting on all qubits contained in the $(d-1)$-hyperplane π_i perpendicular to the Cartesian axis $\widehat{x}_i$. Using an inductive argument over the dimension d one can show that the stabilizer generators specified in Eq. (17) can be constructed from the gauge generators specified in Eqs. (14) and (15).

We now show that the gauge and stabilizer operators defined above commute. Suppose that the edges of $\mathcal{L}$ have unit length and that one of the vertices of $\mathcal{L}$ is at the origin[1]. Every hypercube containing the origin as one of its vertices can be uniquely specified by a string $\mathbf{c} \in \{-1,0,1\}^{\times d}$; the dimensionality of this hypercube is $|\mathbf{c}| = \sum_i |c_i|$. We define the support of a hypercube to be the positions of its non-zero coordinates, i.e., $\operatorname{supp}\mathbf{c} = \{i | c_i \neq 0\}$. We write $\mathbf{a} \subseteq \mathbf{b}$ whenever the non-zero entries of $\mathbf{a}$ match the non-zero entries of $\mathbf{b}$, i.e., $a_i = b_i$ for all $i \in \operatorname{supp}\mathbf{a}$.

Without loss of generality, consider the X-type gauge operator associated with the d-hypercube specified by $\mathbf{a} = (1,\ldots,1)$ and the $(d-3)$-hypercube $\mathbf{c} = (0,0,0,1,\ldots,1)$. This operator has weight three, as it is supported on the following three qubits: $(1,0,0,1,\ldots,1)$, $(0,1,0,1,\ldots,1)$ and $(0,0,1,\ldots,1)$. The only stabilizer generators that have non-trivial overlap with $X(\mathbf{a},\mathbf{c})$ are those associated with neighboring d-hypercubes and those associated with the hyperplanes π_1, π_2 and π_3 that contain the origin. Without loss of generality, consider the Z-type stabilizer associated with d-hypercube $\mathbf{b} = (-1,1,\ldots,1)$. The operators $Z(\mathbf{b})$ and $X(\mathbf{a},\mathbf{c})$ commute, as they overlap on the following two qubits: $(0,1,0,1,\ldots,1)$ and $(0,0,1,\ldots,1)$. In addition, the support of $X(\mathbf{a},\mathbf{c})$ contains exactly two $(d-2)$-hypercubes in each of the relevant hyperplanes, so the hyperplane stabilizers commute with $X(\mathbf{a},\mathbf{c})$.

[1] We only need to consider the neighborhood around the origin as $\mathcal{L}$ is translationally invariant.

Relation to the toric code in $d = 4$ dimensions

Let us now consider a special case of the STC on the four-dimensional hypercubic lattice $\mathcal{L}$. There, the qubits are on faces, local X- and Z-type stabilizer generators are associated with red and blue 4-hypercubes, and gauge generators are associated with pairs comprising a 4-hypercube and an edge contained in the 4-hypercube. The local stabilizer generators have weight 24 and the gauge generators have weight three. We note that the vertices of the 4D hypercubic lattice $\mathcal{L}$ can be colored in green and yellow such that no two vertices sharing an edge have the same color.

We now explain how to obtain the lattice and codes considered in Ref. [7] from $\mathcal{L}$ (the four codes are the same so we need only show that one of them is a gauge-fixing of the 4D STC). The first step is to take the dual of $\mathcal{L}$. This gives a lattice $\mathcal{L}^*$ formed from $\mathcal{L}$ by exchanging vertices, edges, faces, 3-cells and 4-cells respectively with 4-cells, 3-cells, faces, edges, and vertices. It transpires that $\mathcal{L}^*$ is also the 4D hypercubic lattice. Next, we apply a further geometric transformation to $\mathcal{L}^*$ called alternation. We select the blue vertices of $\mathcal{L}^*$ and connect them with new edges if they are part of the same face in $\mathcal{L}^*$. This creates a new tessellation $\mathcal{L}^*_{\rm alt}$ whose vertices correspond to the blue vertices of $\mathcal{L}^*$. The red vertices of $\mathcal{L}^*$ are mapped

to 4-cells in $\mathcal{L}^*_{\text{alt}}$, the faces of $\mathcal{L}^*$ are mapped to edges, the cubic volumes of $\mathcal{L}^*$ are mapped to alternated cubes (tetrahedra) and the 4-hypercubes of $\mathcal{L}^*$ are mapped to 4-hyperoctahedra. The lattice $\mathcal{L}^*_{\text{alt}}$ is a regular tessellation of 4D Euclidean space by 4-hyperoctahedra[2]. The final step is to take the dual of $\mathcal{L}^*_{\text{alt}}$, which gives a tessellation of 4D Euclidean space by octaplexes[3]. We denote the dual of $\mathcal{L}^*_{\text{alt}}$ by $\mathcal{L}_{\text{opx}}$. We illustrate the evolution of a gauge operator through the lattice transformations we have just described in Supplementary Figure 3.

Tracking the faces, 4-hypercubes and edges of $\mathcal{L}$ through the three transformation steps, we find that in $\mathcal{L}_{\text{opx}}$, qubits are placed on 3-cells, X-type stabilizer generators are associated with red vertices, and Z-type gauge operators are associated with pairs comprising an 4-cell and an edge contained in the 4-cell. We note that the vertices of $\mathcal{L}_{\text{opx}}$ can be colored in red, green and yellow such that no two vertices sharing an edge have the same color. In particular, these vertex colors are inherited from the red 4-hypercubes, green vertices and yellow vertices of $\mathcal{L}$. Consequently every Z-type gauge operator in $\mathcal{L}_{\text{opx}}$ is associated with a 4-cell and a GY-edge. In Ref. [7] the authors consider an octaplex tessellation with qubits on 3-cells and vertices 3-colored in, e.g. red, green and yellow. One of their 4D stabilizer toric codes has X-type stabilizer generators associated with red vertices, and Z-type stabilizer generators associated with pairs comprising a 4-cell and a GY edge. This is exactly the construction we just obtained starting from $\mathcal{L}$. Furthermore, we have the inclusions

$$\mathcal{S} \leq \mathcal{S}'_{\text{4DST}} \leq \mathcal{G}, \tag{18}$$

where $\mathcal{S}$ and $\mathcal{G}$ are the stabilizer and gauge groups of the 4D STC, and $\mathcal{S}'_{\text{4DST}}$ is a group generated by stabilizer operators and X-type logical operators of the 4D stabilizer toric code. Therefore, the logical $|\overline{+}\rangle$ state of the 4D stabilizer toric code is also in the code space of the 4D STC, with gauge qubits in a certain state. Moreover, using the procedure of gauge fixing we can map a state in the code space of the 4D STC to the logical $|\overline{+}\rangle$ state of the 4D stabilizer toric code.

We have shown that the 4D STC can be gauge fixed to a code with a transversal 4-qubit control-Z gate. We also expect that the 4D STC will exhibit single-shot QEC and therefore may be an attractive candidate for realizing universal fault-tolerant quantum computation, especially in architectures where non-local connections of qubits are available.

Calculating the number of logical qubits

For simplicity, let us consider the STC on the d-dimensional hypercubic lattice $\mathcal{L}$ with periodic boundary conditions and the linear size L, where L is even. Then, we have

$$|\mathcal{L}_i| = L^d \tbinom{d}{i}. \tag{19}$$

In particular, the number of physical qubits in the d-dimensional STC is

$$N = |\mathcal{L}_{d-2}| = L^d \tbinom{d}{2}. \tag{20}$$

Note that stabilizer generators can be associated with the d-hypercubes and non-contractible $(d-1)$-dimensional hyperplanes within $\mathcal{L}$. Since there are two relations between them, i.e.,

$$\prod_{\delta\in\mathcal{L}_d^R} X(\delta) = \prod_{\delta\in\mathcal{L}_d^B} Z(\delta) = I, \tag{21}$$

we thus obtain that the number of independent generators of the stabilizer group is

$$\log_2 |\mathcal{S}| = |\mathcal{L}_d| + d - 2 = L^d + d - 2. \tag{22}$$

Note that gauge generators, which are associated with d-hypercubes and $(d-3)$-hypercubes, are not independent. Rather, they have to satisfy three types of relations. Relations of the first type, which we call $\mathcal{R}_3$, arise for every $(d-3)$-hypercube $\mu \in \mathcal{L}_{d-3}$ from the following identities

$$\forall\mu \in \mathcal{L}_{d-3}: \prod_{\delta\in\mathcal{L}_d^R:\delta\supset\mu} X(\delta,\mu) = \prod_{\delta\in\mathcal{L}_d^B:\delta\supset\mu} Z(\delta,\mu) = I. \tag{23}$$

Note that not all relations in $\mathcal{R}_3$ are independent. They, as well, have to satisfy certain relations, which we call $\mathcal{R}_4$. They arise for every $(d-4)$-hypercube $\nu \in \mathcal{L}_{d-4}$ from the following identities

$$\forall\nu \in \mathcal{L}_{d-4}: \prod_{\mu\in\mathcal{L}_{d-3}:\mu\supset\nu} \prod_{\delta\in\mathcal{L}_d^R:\delta\supset\mu} X(\delta,\mu) = I, \tag{24}$$

$$\forall\nu \in \mathcal{L}_{d-4}: \prod_{\mu\in\mathcal{L}_{d-3}:\mu\supset\nu} \prod_{\delta\in\mathcal{L}_d^B:\delta\supset\mu} Z(\delta,\mu) = I. \tag{25}$$

But relations $\mathcal{R}_4$ are not independent, and so on. In general, we have

$$|\mathcal{R}_i| = 2|\mathcal{L}_{d-i}| = 2L^d \tbinom{d}{i}. \tag{26}$$

Proper counting of independent relations of the first type gives the following alternating sum

$$\begin{aligned} |\mathcal{R}_3| - |\mathcal{R}_4| + \ldots + (-1)^{d+1}|\mathcal{R}_d| \\ &= 2L^d \sum_{i=3}^{d} (-1)^{i+1} \tbinom{d}{d-i} \\ &= 2L^d \left(\tbinom{d}{d} - \tbinom{d}{d-1} + \tbinom{d}{d-2} \right), \end{aligned} \tag{27}$$

where we use the identity $\sum_{i=0}^{d} (-1)^i \binom{d}{d-i} = 0$. Relations of the second type, which we call $\mathcal{R}'_4$, arise for every d-hypercube $\delta' \in \mathcal{L}_d$ and every $(d-4)$-hypercube $\mu' \in \mathcal{L}_{d-4}$

[2] This tessellation is often called the 16-cell honeycomb.
[3] This tessellation is often called the 24-cell honeycomb.

contained in δ' from the following identities

$$\forall \mathcal{L}_{d-4} \ni \mu' \subset \delta' \in \mathcal{L}_d^R : \prod_{\mu \in \mathcal{L}_{d-3} : \mu' \subset \mu \subset \delta'} X(\delta', \mu) = I, \quad (28)$$

$$\forall \mathcal{L}_{d-4} \ni \mu' \subset \delta' \in \mathcal{L}_d^B : \prod_{\mu \in \mathcal{L}_{d-3} : \mu' \subset \mu \subset \delta'} Z(\delta', \mu) = I. \quad (29)$$

Note that not all relations in $\mathcal{R}'_4$ are independent. They, as well, have to satisfy certain relations, which we call $\mathcal{R}'_5$. They arise for every d-hypercube $\delta' \in \mathcal{L}_d$ and every $(d-5)$-hypercube $\nu' \in \mathcal{L}_{d-5}$ contained in δ' from the following identities

$$\forall \mathcal{L}_{d-5} \ni \nu' \subset \delta' \in \mathcal{L}_d^R : \prod_{\mu' \in \mathcal{L}_{d-4} : \mu' \supset \nu'} \prod_{\mu \in \mathcal{L}_{d-3} : \mu' \subset \mu \subset \delta'} X(\delta', \mu) = I, \quad (30)$$

$$\forall \mathcal{L}_{d-5} \ni \nu' \subset \delta' \in \mathcal{L}_d^B : \prod_{\mu' \in \mathcal{L}_{d-4} : \mu' \supset \nu'} \prod_{\mu \in \mathcal{L}_{d-3} : \mu' \subset \mu \subset \delta'} Z(\delta', \mu) = I. \quad (31)$$

But relations $\mathcal{R}'_5$ are not independent, and so on. In general, we have

$$|\mathcal{R}'_i| = 2^i |\mathcal{L}_d| \tbinom{d}{d-i} = 2^i L^d \tbinom{d}{d-i}. \quad (32)$$

Finally, we arrive at the fact that not all relations $\mathcal{R}'_d$ are independent. In fact, they satisfy one relation for every d-hypercube. Proper counting of independent relations of the second type gives the following alternating sum

$$\begin{aligned} |\mathcal{R}'_4| - |\mathcal{R}'_5| + \ldots + (-1)^d |\mathcal{R}'_d| + (-1)^{d+1} |\mathcal{L}_d| \\ = L_d \left(\sum_{i=4}^{d} (-2)^i \tbinom{d}{d-i} + (-1)^{d+1} \right) \\ = L^d \left(-2^0 \tbinom{d}{0} + 2^1 \tbinom{d}{1} - 2^2 \tbinom{d}{2} + 2^3 \tbinom{d}{3} \right), \end{aligned} \quad (33)$$

where we use the identity $\sum_{i=0}^{d} (-2)^i \tbinom{d}{d-i} = (-1)^d$. Relations of the third type arise for non-contractible $(d-1)$-hyperplanes within $\mathcal{L}$, and thus there are d independent ones. Lastly, relations of the first and second type are not independent, as there are two relations between them—one for all X-type relations and one for all Z-type relations. Once all the independent relations have been properly accounted for, the number of independent generators of the gauge group is

$$\begin{aligned} \log_2 |\mathcal{G}| = 2^3 |\mathcal{L}_d| \tbinom{d}{d-3} - 2L^d \left(\tbinom{d}{d} - \tbinom{d}{d-1} + \tbinom{d}{d-2} \right) \\ - L^d \left(-2^0 \tbinom{d}{0} + 2^1 \tbinom{d}{1} - 2^2 \tbinom{d}{2} + 2^3 \tbinom{d}{3} \right) - d + 2 \\ = L^d \left(2 \tbinom{d}{2} - 1 \right) - d + 2 \end{aligned} \quad (34)$$

By combining Eqs. (20), (22) and (34) we obtain that there are no logical qubits encoded into the STC on the d-dimensional hypercubic lattice with periodic boundary conditions, i.e.,

$$K = N - \frac{1}{2} \left(\log_2 |\mathcal{G}| + \log_2 |\mathcal{S}| \right) = 0. \quad (35)$$

SUPPLEMENTARY NOTE 4: PROOF OF SINGLE-SHOT QEC

Here we prove that single-shot QEC is possible with the 3D STC. For concreteness, we assume that decoding is performed with the single-shot MWPM decoder from the Methods section. Our proof is inspired by the seminal work by Bombín [8] and uses somewhat similar notation. Although our presentation is centered around the 3D STC and the single-shot MWPM decoder, we hope that it provides valuable insights into single-shot QEC in general.

Setting the stage

Let $p : 2^A \to [0,1]$ be a discrete probability distribution over a collection 2^A, which is the power set of some finite set A. We are particularly interested in discrete probability distributions that describe Pauli and measurement errors happening in the system. We say that p is τ-bounded with prefactor c iff for any set $B \subseteq A$ the probability that a set $B' \subseteq A$ drawn according to the probability distribution p contains B is at most $c\tau^{|B|}$, i.e.,

$$\forall B \subseteq A : \sum_{B' \supseteq B} p(B') \leq c\tau^{|B|}. \quad (36)$$

Unless we specify the prefactor explicitly, we assume $c = 1$ by default.

As discussed before, since 3D STC is a CSS code, we can focus our discussion on correcting Pauli X errors, because Pauli Z errors can be corrected analogously. We can describe any stochastic Pauli X noise model as a Pauli X channel, i.e., a completely positive trace-preserving map admitting a Kraus representation

$$\mathcal{N} = \left\{ \sqrt{p_{\mathcal{N}}(N)} N \right\}_N, \quad (37)$$

where $p_{\mathcal{N}}$ is a discrete probability distribution over Pauli X operators, i.e., $\sum_N p_{\mathcal{N}}(N) = 1$. We denote the collection of all Pauli X channels by

$$\mathbb{P}^X = \{ \mathcal{N} \mid \mathcal{N} \text{ is a Pauli } X \text{ channel} \}. \quad (38)$$

We say that $\mathcal{N} \in \mathbb{P}^X$ is τ-bounded with prefactor c iff the corresponding probability distribution $p_{\mathcal{N}}$ is τ-bounded with prefactor c, i.e.,

$$\forall N : \sum_{N' \supseteq N} p_{\mathcal{N}}(N') \leq c\tau^{|N|}. \quad (39)$$

We remark that in Eq. (39) as well as in the rest of the article we write N and M to denote both the Pauli X operators themselves, as well as their support, however it should be clear from the context what we mean. For instance, ∂N denotes the stabilizer syndrome of N, $N \cup M$ and $N \cap M$ denote, respectively, the union and

intersection of the supports of N and M, NM denotes either the Pauli X operator, which is the product of N and M, or its support.

For any two channels $\mathcal{N} = \{\sqrt{p_{\mathcal{N}}(N)}N\}_N$ and $\mathcal{M} = \{\sqrt{p_{\mathcal{M}}(M)}M\}_M$ we define their composition as

$$\mathcal{N} \circ \mathcal{M} = \left\{\sqrt{p_{\mathcal{N}}(N)p_{\mathcal{M}}(M)}NM\right\}_{N,M}. \quad (40)$$

Lastly, for any two collections of channels $\mathbb{N}$ and $\mathbb{M}$, we define their composition as the following collection of channels

$$\mathbb{N} \circ \mathbb{M} = \{\mathcal{N} \circ \mathcal{M} \mid \mathcal{N} \in \mathbb{N}, \mathcal{M} \in \mathbb{M}\}. \quad (41)$$

Let $G = (V, E)$ be a hypergraph consisting of vertices V and hyperedges E. Note that a hypergraph is a generalization of a graph, in which a hyperedge is a non-empty subset of vertices. For any subset of vertices $V' \subseteq V$ we can construct a new hypergraph $G' = (V \setminus V', \{e \setminus V' | e \in E\})$ by removing all the vertices V' from the hypergraph G. We say that a subset A of hyperedges E is connected if a subhypergraph of G that includes all the hyperedges in A is connected. For any $A \subseteq E$ we can find a unique decomposition $A = \bigcup_i A_i$ in terms of its connected components, where each A_i is a maximal connected subset of A. We say that a subset B of hyperedges E is a connected cover of A iff B contains A and every connected component B_i of B intersects A, i.e., $B \supseteq A$ and $B_i \cap A \neq \emptyset$. We denote by $\mathfrak{C}(s, A)$ the collection of connected covers of A that contain s hyperedges. Lastly, for any $A, B \subseteq E$ we define $A^{\cap B}$ to be the union of all connected components A_i of A that intersect B, namely

$$A^{\cap B} = \bigcup_{i: A_i \cap B \neq \emptyset} A_i. \quad (42)$$

Note that if $A \supseteq B$, then $A^{\cap B}$ is a connected cover of B of size $s = |A^{\cap B}|$, i.e., $A^{\cap B} \in \mathfrak{C}(s, A)$.

The following lemma will be useful in bounding the number of connected covers.

Lemma 2 (connected covers)**.** *Let $A \subseteq E$ be a subset of hyperedges in a finite hypergraph $G = (V, E)$. Then, the number of connected covers of A that contain s hyperedges satisfies*

$$|\mathfrak{C}(s, A)| \leq \frac{(\mathfrak{e}z)^s}{\mathfrak{e}z^{|A|}}, \qquad z = \max_{e \in E} \sum_{v \in e} (\deg v - 1), \quad (43)$$

where $\mathfrak{e}$ is Euler's number and $\deg v$ denotes the degree of a vertex v of G.

Proof. Let $G' = (V', E')$ be a graph, whose vertices correspond to the hyperedges of G, i.e., $V' = E$. We connect two vertices in G' with an edge iff the corresponding hyperedges in G are incident to the same vertex. Note that maximum degree of the graph G' is then z. Let $A' \subseteq V'$ be the subset of vertices corresponding to the subset of hyperedges $A \subseteq E$. Then, the task of counting the number of connected covers of A in G is equivalent to the cluster counting problem in Lemma 5 in Ref. [9] for A' in G'. We thus obtain the upper bound $\mathfrak{e}^{|A|-1}(\mathfrak{e}z)^{s-|A|}$. □

3D STC lattice

In the rest of this section we focus our attention on the 3D STC defined on some lattice $\mathcal{L}$. Using the notions from the Methods section, let G_{mea} and G_{qub} be the measurement and qubit graphs associated with $\mathcal{L}$, and let V'_{mea} and V'_{qub} be the sets of boundary vertices of G_{mea} and G_{qub}, respectively. Recall that $V'_{\text{mea}} \supseteq V'_{\text{qub}}$ as $V_{\text{mea}} \supseteq V_{\text{qub}}$. When we talk about connected components and connected covers we always consider them within a hypergraph $G_{\mathcal{L}} = (V_{\mathcal{L}}, E_{\mathcal{L}})$, which is obtained by taking the union of G_{mea} and G_{qub}, and then removing all the boundary vertices, i.e.,

$$G_{\mathcal{L}} = (V_{\text{mea}} \setminus V'_{\text{mea}}, \{e \setminus V'_{\text{mea}} | e \in E_{\text{mea}} \cup E_{\text{qub}}\}). \quad (44)$$

Note that $G_{\mathcal{L}}$ is a hypergraph since every edge in $E_{\text{mea}} \cup E_{\text{qub}}$ that is incident to exactly one boundary vertex in V'_{mea} is replaced by the other vertex it is incident to. The hyperedges $E_{\mathcal{L}}$ correspond to the measurement outcomes and Pauli X errors. The vertices $V_{\mathcal{L}}$ and $V_{\text{qub}} \setminus V'_{\text{qub}}$ correspond to, respectively, locations where the Gauss law has to be satisfied and the Z-type stabilizers. Let $\Delta_{\mathcal{L}}$ denote the maximum vertex degree of $G_{\mathcal{L}}$, i.e.,

$$\Delta_{\mathcal{L}} = \max_{v \in V_{\mathcal{L}}} \deg v. \quad (45)$$

Let Δ_{qub} and Σ_{qub} be the maximum vertex degree for interior vertices of G_{qub} and the sum of the degrees of all the boundary vertices in V'_{qub}, namely

$$\Delta_{\text{qub}} = \max_{v \in V_{\text{qub}} \setminus V'_{\text{qub}}} \deg v, \quad (46)$$

$$\Sigma_{\text{qub}} = \sum_{v \in V'_{\text{qub}}} \deg v. \quad (47)$$

In order to prove the existence of a non-zero threshold for the 3D STC, we need to assume that we have a family of lattices parametrized by a positive integer L. The integer L provides a lower bound on the shortest distance between any two different boundary vertices in the qubit graph G_{qub} associated with the lattice $\mathcal{L}$. For such a lattice family, we require that $\Delta_{\mathcal{L}}$ and Δ_{qub} are bounded by some constants, and that Σ_{qub} scales polynomially in L. Moreover, in order to prove the main result, (single-shot QEC) Theorem 1, we need the following assumption about the lattice $\mathcal{L}$ and the associated hypergraph $G_{\mathcal{L}}$ to hold.

- **Confinement of the flux.**—For any Z-type gauge flux φ there exists a Pauli X operator M, such that the stabilizer syndromes of M and φ are

the same, i.e., $\partial_S M = \delta_S \varphi$, and M is of comparable size to φ, i.e.,

$$|M| \leq c_{\text{flu}} |\varphi|. \tag{48}$$

We remark that the assumption about confinement of the flux is analogous to the confining property specified in Definition 16 in Ref. [8]. Moreover, for the lattice $\mathcal{L}^*_{\text{cub}}$ of linear length L, which we describe in the Results section, the constants are

$$\Delta_{\mathcal{L}} = 20, \tag{49}$$
$$\Delta_{\text{qub}} = 12, \tag{50}$$
$$\Sigma_{\text{qub}} = 4L^2 + 6L + 2, \tag{51}$$
$$c_{\text{flu}} = 1/4. \tag{52}$$

Error correction with and without measurement errors

We now revisit the question of how to perform QEC, which we already discussed at length in the Methods section. Let us start with the case of ideal error correction, when there are no measurement errors. To simplify the notation, in the rest of this section we write ∂ to denote both δ_S and ∂_S, however it should be clear from the context what we mean. First, to diagnose Pauli X errors we measure the set of Z-type gauge operators in the gauge group $\mathcal{G}$ of the 3D STC, which correspond to the edges of the measurement graph G_{mea}. Let φ be the flux, i.e., the set of gauge operators returning -1 measurement outcomes. Knowing the flux φ we can infer the measurement outcomes for the set of Z-type stabilizer operators in the stabilizer group $\mathcal{S}$ of the 3D STC, which correspond to the vertices of the qubit graph G_{qub}. Namely, if σ denotes the stabilizer syndrome, i.e., the set of stabilizer operators returning -1 measurement outcomes, then we have $\sigma = \partial\varphi$. Lastly, knowing the stabilizer syndrome σ and using the ideal MWPM decoder we can find the minimum-weight Pauli X recovery operator R_σ, such that $\partial R_\sigma = \sigma$.

We can succinctly describe ideal error correction by the following channel

$$\mathcal{R}_0 = \left\{R_{\partial\varphi}\Pi^{\mathcal{G}}_{\varphi}\right\}_{\varphi} = \left\{R_\sigma \sum_{\varphi:\partial\varphi=\sigma} \Pi^{\mathcal{G}}_{\varphi}\right\}_{\sigma} = \left\{R_\sigma \Pi^{\mathcal{S}}_{\sigma}\right\}_{\sigma}, \tag{53}$$

where $\Pi^{\mathcal{G}}_{\varphi}$ and $\Pi^{\mathcal{S}}_{\sigma} = \sum_{\varphi:\partial\varphi=\sigma} \Pi^{\mathcal{G}}_{\varphi}$ are the projection operators onto the subspaces with the given flux φ and stabilizer syndrome σ, respectively. We refer to $\mathcal{R}_0$ as the ideal MWPM decoding channel.

Let $\mathcal{N} = \{\sqrt{p_{\mathcal{N}}(N)}N\}_N \in \mathbb{P}^X$ and consider the following composite channel

$$\begin{aligned} \mathcal{R}_0 \circ \mathcal{N} \circ \Pi^{\mathcal{S}}_0 &= \left\{\sqrt{p_{\mathcal{N}}(N)} R_\sigma \Pi^{\mathcal{S}}_{\sigma} N \Pi^{\mathcal{S}}_0\right\}_{\sigma,N} \\ &= \left\{\sqrt{p_{\mathcal{N}}(N)} R_{\partial N} N \Pi^{\mathcal{S}}_0\right\}_{N}, \end{aligned} \tag{54}$$

where $\Pi^{\mathcal{S}}_0$ is the projection operator onto the 3D STC code subspace, i.e., the subspace with the trivial stabilizer syndrome $\sigma = 0$. Note that in Eq. (54) we use the fact that for any two stabilizer syndromes σ and σ', and any Pauli X operator N we have $\Pi^{\mathcal{S}}_{\sigma}\Pi^{\mathcal{S}}_{\sigma'} = \delta_{\sigma,\sigma'}\Pi^{\mathcal{S}}_{\sigma'}$ and $\Pi^{\mathcal{S}}_{\sigma} N = N\Pi^{\mathcal{S}}_{\sigma+\partial N}$, where $\delta_{\sigma,\sigma'}$ denotes the Kronecker delta. Then, we define

$$\text{fail}(\mathcal{N}) = \sum_{N: R_{\partial N} N \notin \mathcal{G}} p_{\mathcal{N}}(N) \tag{55}$$

to be the probability that the channel $\mathcal{R}_0 \circ \mathcal{N} \circ \Pi^{\mathcal{S}}_0$ implements any non-trivial logical Pauli operator. We refer to fail($\mathcal{N}$) as the failure probability of the ideal MWPM decoding channel for the Pauli X channel $\mathcal{N}$ or, in short, the failure probability for $\mathcal{N}$. Moreover, we show the following lemma about the ideal MWPM decoding channel $\mathcal{R}_0$.

Lemma 3. *(decoding failure) Let* $\mathcal{N} = \{\sqrt{p_{\mathcal{N}}(N)}N\}_N$ *be a* τ*-bounded Pauli* X *channel. If* $\tau < \tau^*$*, where* $\tau^* = (2(\Delta_{\text{qub}} - 1))^{-2}$*, then the failure probability of the ideal MWPM decoding channel* $\mathcal{R}_0$ *for* $\mathcal{N}$ *satisfies*

$$\text{fail}(\mathcal{N}) \leq f(\tau), \qquad f(\tau) = \frac{\Sigma_{\text{qub}}\tau^*}{(\tau^*)^{1/2} - \tau^{1/2}} \left(\frac{\tau}{\tau^*}\right)^{L/2}. \tag{56}$$

We remark that for the family of lattices considered in the Methods section, (decoding failure) Lemma 3 immediately implies the existence of $\tau^* > 0$, such that $\lim_{L\to\infty} \text{fail}(\mathcal{N}) = 0$ for $\tau < \tau^*$, i.e., that there exists a non-zero threshold τ^* for the 3D STC with the ideal MWPM decoder and the τ-bounded Pauli X channel $\mathcal{N}$. Thus, (decoding failure) Lemma 3 can be viewed as a special case of more general results establishing non-zero thresholds for certain families of quantum low-density parity-check codes in Refs. [10, 11]. Plugging Eq. (50) into the expression for τ^* in Lemma 3, we find that for the 3D STC defined on the lattice $\mathcal{L}^*_{\text{cub}}$ we have $\tau^* = 1/484 \approx 0.21\%$, which is approximately one-fifth of the numerical value (the $t = 0$ data-point in Fig. 8a) observed in simulations.

Proof. Let $\Lambda(i)$ denote the set of simple paths[4] of length i in the qubit graph G_{qub}, such that each path contains exactly two different vertices in V'_{qub}, where it starts and ends. Roughly speaking, $\Lambda(i)$ contains paths connecting different boundaries of $\mathcal{L}$. By definition, $\Lambda(i) = \emptyset$ for any $i < L$. Let us now assume that $i \geq L$. Since any path $\lambda \in \Lambda(i)$ starts at some boundary vertex in G_{qub}, the first edge of λ can be chosen in Σ_{qub} ways. Moreover, every following edge of λ can be chosen in at most $\Delta_{\text{qub}} - 1$

[4] A simple path is a path in a graph that does not have repeating vertices.

ways, as the path λ cannot backtrack. Thus, we arrive at the following upper bound

$$|\Lambda(i)| \leq \Sigma_{\mathrm{qub}}(\Delta_{\mathrm{qub}}-1)^{i-1}. \tag{57}$$

We proceed by first proving the following inequality

$$\begin{aligned} \mathrm{fail}(\mathcal{N}) &= \sum_{N:R_{\partial N}N\notin\mathcal{G}} p_{\mathcal{N}}(N) \\ &\leq \sum_{i\geq L}\sum_{\lambda\in\Lambda(i)}\sum_{N'\subseteq\lambda:|N'|\geq\frac{i}{2}}\sum_{N\supseteq N'} p_{\mathcal{N}}(N), \end{aligned} \tag{58}$$

where in the summation on the right-hand side we treat N and N' as the subsets of qubits supporting the corresponding Pauli X operators rather than those operators themselves. Let N be a Pauli X error, for which $p_{\mathcal{N}}(N)$ appears on the left-hand side of the inequality. Since $R_{\partial N}N \notin \mathcal{G}$, the support of $R_{\partial N}N$ is guaranteed to contain some path $\lambda \in \Lambda(i)$ for some $i \geq L$, i.e., $\lambda \subseteq R_{\partial N}N$. We now show that the term $p_{\mathcal{N}}(N)$ appears on the right-hand side for $N' = N\cap\lambda$. Let $R' = R_{\partial N}\cap\lambda$. Then, $\partial N' = \partial R'$ and $|\lambda| = |N'| + |R'|$. Since $R_{\partial N}$ is the minimum-weight recovery operator with the stabilizer syndrome ∂N, we also have $|R'| \leq |N'|$; otherwise, $N'R'R_{\partial N}$ would be a recovery operator with the stabilizer syndrome ∂N of weight smaller than $R_{\partial N}$. Thus, $|N'| \geq |\lambda|/2 = i/2$. This, in turn, implies that the term $p_{\mathcal{N}}(N)$ appears on the right-hand side and, subsequently, establishes the inequality in Eq. (58), as all the terms in the inequality are non-negative.

Using the fact that $p_{\mathcal{N}}$ is τ-bounded and Eq. (57) we obtain

$$\begin{aligned} &\sum_{i\geq L}\sum_{\lambda\in\Lambda(i)}\sum_{N'\subseteq\lambda:|N'|\geq\frac{i}{2}}\sum_{N\supseteq N'} p_{\mathcal{N}}(N) \\ &\quad\leq \sum_{i\geq L}\sum_{\lambda\in\Lambda(i)}\sum_{N'\subseteq\lambda:|N'|\geq\frac{i}{2}} \tau^{|N'|} \\ &\quad\leq \sum_{i\geq L}\sum_{\lambda\in\Lambda(i)} 2^{i-1}\tau^{i/2} \\ &\quad\leq \sum_{i\geq L}\Sigma_{\mathrm{qub}}(\Delta_{\mathrm{qub}}-1)^{i-1}2^{i-1}\tau^{i/2} \\ &\quad\leq \frac{\Sigma_{\mathrm{qub}}\left(2(\Delta_{\mathrm{qub}}-1)\tau^{1/2}\right)^L}{2(\Delta_{\mathrm{qub}}-1)\left(1-2(\Delta_{\mathrm{qub}}-1)\tau^{1/2}\right)}, \end{aligned} \tag{59}$$

leading to $\mathrm{fail}(\mathcal{N}) \leq f(\tau)$. □

Now, we consider the case of error correction with imperfect measurements. As before, we diagnose Pauli X errors by measuring Z-type gauge operators. This time, however, we do not learn the flux φ; rather, we register the measurement outcome $\zeta = \varphi + \mu$, where μ is the measurement error, i.e., the set of gauge operators whose corresponding measurement outcomes have flipped. We assume that the measurement error μ is independent of the flux φ. We proceed by finding the minimum-weight measurement error estimate $\widehat{\mu}$, such that $\partial_R\widehat{\mu} = \partial_R\zeta$. Knowing the measurement outcome ζ and the measurement error estimate $\widehat{\mu}$ we can infer an estimate $\widehat{\sigma}$ of the stabilizer syndrome $\sigma = \partial\varphi$, namely $\widehat{\sigma} = \partial(\varphi+\mu+\widehat{\mu})$. Lastly, using the ideal MWPM decoder we can find the minimum-weight Pauli X recovery operator $R_{\widehat{\sigma}}$, such that $\partial R_{\widehat{\sigma}} = \widehat{\sigma}$.

We can capture error correction with imperfect measurements with the following channel

$$\begin{aligned} \mathcal{R} &= \left\{\sqrt{p_{\mathcal{R}}(\mu)}R_{\partial(\varphi+\mu+\widehat{\mu})}\Pi_{\varphi}^{\mathcal{G}}\right\}_{\mu,\varphi} \\ &= \left\{\sqrt{p_{\mathcal{R}}(\mu)}R_{\sigma+\partial(\mu+\widehat{\mu})}\sum_{\varphi:\partial\varphi=\sigma}\Pi_{\varphi}^{\mathcal{G}}\right\}_{\mu,\sigma} \\ &= \left\{\sqrt{p_{\mathcal{R}}(\mu)}R_{\sigma+\partial(\mu+\widehat{\mu})}\Pi_{\sigma}^{\mathcal{S}}\right\}_{\mu,\sigma}, \end{aligned} \tag{60}$$

where $p_{\mathcal{R}}(\mu)$ is the probability that the measurement error μ occurs and $\sum_\mu p_{\mathcal{R}}(\mu) = 1$. For brevity, we also refer to $\mathcal{R}$ as the single-shot MWPM decoding channel.

Lastly, we introduce $\mathbb{R}_\eta$ to be a class of channels consisting of all the single-shot MWPM decoding channels, for which the measurement error probability distributions are η-bounded, i.e.,

$$\begin{aligned} \mathbb{R}_\eta = \{&\mathcal{R}\text{ is a single-shot MWPM} \\ &\text{decoding channel} \mid p_{\mathcal{R}}\text{ is }\eta\text{-bounded}\}. \end{aligned} \tag{61}$$

Note that the ideal MWPM decoding channel $\mathcal{R}_0$ can be viewed as the single-shot MWPM decoding channel with $p_{\mathcal{R}_0}(\mu) = \delta_{\mu,0}$. In that case, the measurement error probability distribution $p_{\mathcal{R}_0}$ is 0-bounded and thus $\mathcal{R}_0 \in \mathbb{R}_0$.

More on channels

Let $\mathcal{N} = \{\sqrt{p_{\mathcal{N}}(N)}N\}_N \in \mathbb{P}^X$ and $\mathcal{R}_0$ be the ideal MWPM decoding channel defined in Eq. (53). We equivalently express $\mathcal{N}$ in the following decoder-dependent form

$$\mathcal{N} = \{\sqrt{p_{\mathcal{N}}(N)}R_{\partial N}(R_{\partial N}N)\}_N. \tag{62}$$

Note that the recovery operator $R_{\partial N}$ has the same stabilizer syndrome as N, i.e., $\partial R_{\partial N} = \partial N$, whereas $R_{\partial N}N$ forms a logical (possibly trivial) Pauli operator, i.e., $R_{\partial N}N \in \mathcal{Z}(\mathcal{S})$. Then, we define the $\mathcal{R}_0$-dependent channel $\overline{\mathcal{N}}$ for the Pauli X channel $\mathcal{N}$ as follows

$$\overline{\mathcal{N}} = \{\sqrt{p_{\mathcal{N}}(N)}R_{\partial N}\}_N = \left\{\sqrt{p_{\overline{\mathcal{N}}}(N')}N'\right\}_{N'}, \tag{63}$$

where we group the same Pauli X terms and introduce

$$p_{\overline{\mathcal{N}}}(N') = \sum_{N:R_{\partial N}=N'} p_{\mathcal{N}}(N). \tag{64}$$

By definition, we have $\mathrm{fail}(\overline{\mathcal{N}}) = 0$.

For completeness, we reprove the following lemma.

Lemma 4 (Lemma 1 in Ref. [8])**.** *For any two $\mathcal{N},\mathcal{M} \in \mathbb{P}^X$ we have*

$$\overline{\mathcal{N}\circ\mathcal{M}} = \overline{\overline{\mathcal{N}}\circ\overline{\mathcal{M}}}, \tag{65}$$

$$\mathrm{fail}(\mathcal{N}\circ\mathcal{M}) \le \mathrm{fail}(\mathcal{N}) + \mathrm{fail}(\mathcal{M}) + \mathrm{fail}(\overline{\mathcal{N}}\circ\overline{\mathcal{M}}), \tag{66}$$

$$\mathrm{fail}(\overline{\mathcal{N}}\circ\overline{\mathcal{M}}) \le \mathrm{fail}(\mathcal{N}) + \mathrm{fail}(\mathcal{M}) + \mathrm{fail}(\mathcal{N}\circ\mathcal{M}). \tag{67}$$

We remark that although we define the $\mathcal{R}_0$-dependent channel and the failure probability in terms of the ideal MWPM decoding channel, Lemma 4 also holds for other decoding channels (with appropriately modified definitions).

Proof. To prove Eq. (65), we show that $p_{\overline{\mathcal{N}\circ\mathcal{M}}}(P) = p_{\overline{\overline{\mathcal{N}}\circ\overline{\mathcal{M}}}}(P)$ for any Pauli X operator P. Namely,

$$p_{\overline{\mathcal{N}\circ\mathcal{M}}}(P) = \sum_{P':R_{\partial P'}=P} p_{\mathcal{N}\circ\mathcal{M}}(P') \tag{68}$$

$$= \sum_{\substack{N,M:\\ R_{\partial(NM)}=P}} p_{\mathcal{N}}(N)p_{\mathcal{M}}(M) \tag{69}$$

$$= \sum_{\substack{N',M':\\ R_{\partial(N'M')}=P}} \sum_{N:R_{\partial N}=N'} p_{\mathcal{N}}(N) \sum_{M:R_{\partial M}=M'} p_{\mathcal{M}}(M) \tag{70}$$

$$= \sum_{\substack{N',M':\\ R_{\partial(N'M')}=P}} p_{\overline{\mathcal{N}}}(N')p_{\overline{\mathcal{M}}}(M') \tag{71}$$

$$= \sum_{P':R_{\partial P'}=P} p_{\overline{\mathcal{N}}\circ\overline{\mathcal{M}}}(P') = p_{\overline{\overline{\mathcal{N}}\circ\overline{\mathcal{M}}}}(P), \tag{72}$$

where in Eq. (70) we group Pauli terms N's and M's according to their syndromes $\partial N'$ and $\partial M'$, respectively. Then, we proceed by writing the failure probabilities for $\mathcal{N}\circ\mathcal{M}$ and $\overline{\mathcal{N}}\circ\overline{\mathcal{M}}$ as follows

$$\begin{aligned}\mathrm{fail}(\mathcal{N}\circ\mathcal{M}) &= \sum_{P:R_{\partial P}P\notin\mathcal{G}} p_{\mathcal{N}\circ\mathcal{M}}(P)\\ &= \sum_{\substack{N,M:\\ R_{\partial(NM)}NM\notin\mathcal{G}}} p_{\mathcal{N}}(N)p_{\mathcal{M}}(M),\end{aligned} \tag{73}$$

$$\begin{aligned}\mathrm{fail}(\overline{\mathcal{N}}\circ\overline{\mathcal{M}}) &= \sum_{P:R_{\partial P}P\notin\mathcal{G}} p_{\overline{\mathcal{N}}\circ\overline{\mathcal{M}}}(P)\\ &= \sum_{\substack{N,M:\\ R_{\partial(NM)}R_{\partial N}R_{\partial M}\notin\mathcal{G}}} p_{\mathcal{N}}(N)p_{\mathcal{M}}(M).\end{aligned} \tag{74}$$

Since $R_{\partial(NM)}NM = (R_{\partial N}N)(R_{\partial M}M)(R_{\partial(NM)}R_{\partial M}R_{\partial M})$, then the condition $R_{\partial(NM)}NM \notin \mathcal{G}$ implies that $R_{\partial N}N \notin \mathcal{G}$ or $R_{\partial M}M \notin \mathcal{G}$ or $R_{\partial(NM)}R_{\partial M}R_{\partial M} \notin \mathcal{G}$, Thus,

$$\begin{aligned}\mathrm{fail}(\mathcal{N}\circ\mathcal{M}) &= \sum_{\substack{N,M:\\ R_{\partial(NM)}NM\notin\mathcal{G}}} p_{\mathcal{N}}(N)p_{\mathcal{M}}(M)\\ &\le \sum_{\substack{N,M:\\ R_{\partial N}N\notin\mathcal{G}}} p_{\mathcal{N}}(N)p_{\mathcal{M}}(M) + \sum_{\substack{N,M:\\ R_{\partial M}M\notin\mathcal{G}}} p_{\mathcal{N}}(N)p_{\mathcal{M}}(M)\\ &+ \sum_{\substack{N,M:\\ R_{\partial(NM)}R_{\partial N}R_{\partial M}\notin\mathcal{G}}} p_{\mathcal{N}}(N)p_{\mathcal{M}}(M)\\ &= \mathrm{fail}(\mathcal{N}) + \mathrm{fail}(\mathcal{M}) + \mathrm{fail}(\overline{\mathcal{N}}\circ\overline{\mathcal{M}})\end{aligned} \tag{75}$$

and we obtain the inequality in Eq. (66). Similarly, the condition $R_{\partial(NM)}R_{\partial N}R_{\partial M} \notin \mathcal{G}$ implies that $R_{\partial N}N \notin \mathcal{G}$ or $R_{\partial M}M \notin \mathcal{G}$ or $R_{\partial(NM)}NM \notin \mathcal{G}$, and we can establish the inequality in Eq. (67). □

We say that two Pauli X channels $\mathcal{N}$ an $\mathcal{M}$ are $\mathcal{R}_0$-equivalent and write $\mathcal{N}\sim\mathcal{M}$ iff their corresponding $\mathcal{R}_0$-dependent channels $\overline{\mathcal{N}}$ and $\overline{\mathcal{M}}$ are the same. We emphasize that for any two $\mathcal{R}_0$-equivalent $\mathcal{N},\mathcal{M} \in \mathbb{P}^X$ their resulting stabilizer syndrome distributions are the same, however terms in their Kraus representations might differ by some X-type gauge or logical Pauli operators. Also note that $\mathcal{N}\sim\overline{\mathcal{N}}$, as $\overline{\overline{\mathcal{N}}} = \overline{\mathcal{N}}$.

Lastly, we introduce $\mathbb{N}_{\tau,\epsilon}$ to be a class comprising all the Pauli X channels that are $\mathcal{R}_0$-equivalent to any τ-bounded Pauli X channel and whose failure probability is at most ϵ, i.e.,

$$\begin{aligned}\mathbb{N}_{\tau,\epsilon} = \{\mathcal{N}\in\mathbb{P}^X \mid \mathrm{fail}(\mathcal{N}) \le \epsilon \text{ and there exists}\\ \text{a } \tau\text{-bounded } \mathcal{M}\in\mathbb{P}^X \text{ satisfying } \mathcal{M}\sim\mathcal{N}\}.\end{aligned} \tag{76}$$

We finish this section with the following lemma about composing channels from the class $\mathbb{N}_{\tau,\epsilon}$.

Lemma 5 (composition)**.** *For sufficiently small τ_1 and τ_2 there exist τ' and ϵ', such that the following inclusion holds*

$$\mathbb{N}_{\tau_1,\epsilon_1}\circ\mathbb{N}_{\tau_2,\epsilon_2} \subseteq \mathbb{N}_{\tau',\epsilon'}. \tag{77}$$

In particular, we can have $\tau' = \tau_1+\tau_2$ and $\epsilon' = \epsilon_1 + \epsilon_2 + f(\tau_1) + f(\tau_2) + f(\tau_1+\tau_2)$, where $f(\cdot)$ is the upper bound specified in (decoding failure) Lemma 3, provided that $\tau_1+\tau_2 < (2(\Delta_{\mathrm{qub}}-1))^{-2}$.

Proof. Let $\mathcal{N}\in\mathbb{N}_{\tau_1,\epsilon_1}$ and $\mathcal{M}\in\mathbb{N}_{\tau_2,\epsilon_2}$. Let $\mathcal{N}' = \{\sqrt{p_{\mathcal{N}'}(N')}N'\}_{N'}$ and $\mathcal{M}' = \{\sqrt{p_{\mathcal{M}'}(M')}M'\}_{M'}$ be two Pauli X channels, which are $\mathcal{R}_0$-equivalent to $\mathcal{N}$ and $\mathcal{M}$ and which are τ_1-bounded and τ_2-bounded, respectively. Using Eq. (65) we have

$$\overline{\mathcal{N}'\circ\mathcal{M}'} = \overline{\overline{\mathcal{N}'}\circ\overline{\mathcal{M}'}} = \overline{\overline{\mathcal{N}}\circ\overline{\mathcal{M}}} = \overline{\mathcal{N}\circ\mathcal{M}}, \tag{78}$$

thus the composite channel $\mathcal{N}'\circ\mathcal{M}'$ is $\mathcal{R}_0$-equivalent to $\mathcal{N}\circ\mathcal{M}$. We can straightforwardly show that $\mathcal{N}'\circ\mathcal{M}'$ is

$(\tau_1+\tau_2)$-bounded, namely

$$\begin{aligned}
\sum_{P'\supseteq P} p_{\mathcal{N}'\circ\mathcal{M}'}(P') &= \sum_{\substack{N',M':\\ N'M'\supseteq P}} p_{\mathcal{N}'}(N')p_{\mathcal{M}'}(M') \\
&= \sum_{P'\subseteq P} \sum_{\substack{N'\supseteq P',M'\supseteq P\setminus P':\\ N'\cap M'\cap P=\emptyset}} p_{\mathcal{N}'}(N')p_{\mathcal{M}'}(M') \\
&\leq \sum_{P'\subseteq P}\sum_{N'\supseteq P'} p_{\mathcal{N}'}(N') \sum_{M'\supseteq P\setminus P'} p_{\mathcal{M}'}(M') \\
&\leq \sum_{P'\subseteq P} \tau_1^{|P'|}\tau_2^{|P\setminus P'|} = (\tau_1+\tau_2)^{|P|}.
\end{aligned} \tag{79}$$

This allows us to conclude that $\mathcal{N}\circ\mathcal{M}\in\mathbb{N}_{\tau',*}$.

Using the inequalities from Lemma 4 and applying (decoding failure) Lemma 3 to $\mathcal{N}'$, $\mathcal{M}'$ and $\mathcal{N}'\circ\mathcal{M}'$ we obtain

$$\begin{aligned}
\mathrm{fail}(\mathcal{N}\circ\mathcal{M}) &\leq \mathrm{fail}(\mathcal{N})+\mathrm{fail}(\mathcal{M})+\mathrm{fail}(\overline{\mathcal{N}}\circ\overline{\mathcal{M}}) \\
&= \epsilon_1+\epsilon_2+\mathrm{fail}(\overline{\mathcal{N}'}\circ\overline{\mathcal{M}'}) \\
&\leq \epsilon_1+\epsilon_2+\mathrm{fail}(\mathcal{N}')+\mathrm{fail}(\mathcal{M}')+\mathrm{fail}(\mathcal{N}'\circ\mathcal{M}') \\
&\leq \epsilon_1+\epsilon_2+f(\tau_1)+f(\tau_2)+f(\tau_1+\tau_2),
\end{aligned} \tag{80}$$

which allows us to conclude that $\mathcal{N}\circ\mathcal{M}\in\mathbb{N}_{*,\epsilon'}$. □

Single-shot QEC theorem

Theorem 1. *For sufficiently small η and τ there exist τ' and ϵ' satisfying*

$$\lim_{\eta\to 0}\tau' = 0, \qquad \lim_{L\to\infty}\epsilon' = \epsilon, \tag{81}$$

such that the following inclusion holds

$$\mathbb{R}_\eta\circ\mathbb{N}_{\tau,\epsilon}\circ\Pi_0^{\mathcal{S}} \subseteq \mathbb{N}_{\tau',\epsilon'}\circ\Pi_0^{\mathcal{S}}. \tag{82}$$

In particular, we can have $\tau' = 2^{2t-1}(\mathfrak{e}\eta^r)^t(\Delta_{\mathcal{L}}-1)^{t-1}$ and $\epsilon' = \epsilon+f(\tau)+f(\tau+\tau')$, where $\mathfrak{e}$ denotes Euler's number, $t=1+c_{\mathrm{flu}}^{-1}$, $r=(2+2c_{\mathrm{flu}})^{-1}$ and $f(\cdot)$ is the upper bound specified in (decoding failure) Lemma 3, provided that $\eta<(\mathfrak{e}-1)^{1/r}(4\mathfrak{e}^2(\Delta_{\mathcal{L}}-1))^{-1/r}$ and $\tau+\tau'\leq(2(\Delta_{\mathrm{qub}}-1))^{-2}$.

Importantly, (single-shot QEC) Theorem 1 says that the parameter τ' of the residual noise present in the system after performing one round of error correction with imperfect measurements can be made arbitrarily small only by reducing the parameter η of the single-shot MWPM decoding channel, i.e., $\lim_{\eta\to0}\tau'=0$. Moreover, the failure probability ϵ' for the residual noise increases by at most $f(\tau)+f(\tau+\tau')$ compared to ϵ and that increment can be made arbitrarily small just by increasing the linear size L of the system, i.e., $\lim_{L\to\infty}(f(\tau)+f(\tau+\tau'))=0$. This establishes that the single-shot MWPM decoder is fault-tolerant and, subsequently, single-shot QEC is possible with the 3D STC.

Proof. Let $\mathcal{R} = \{\sqrt{p_{\mathcal{R}}(\mu)}R_{\partial(\varphi+\mu+\widehat{\mu})}\Pi_\varphi^{\mathcal{G}}\}_{\mu,\varphi}\in\mathbb{R}_\eta$ and $\mathcal{N}=\{\sqrt{p_{\mathcal{N}}(N)}N\}_N\in\mathbb{N}_{\tau,\epsilon}$. Let $\mathcal{M}\in\mathbb{P}^X$ be defined as follows

$$\begin{aligned}
\mathcal{M} &= \{\sqrt{p_{\mathcal{R}}(\mu)p_{\mathcal{N}}(N)}R_{\partial N+\partial(\mu+\widehat{\mu})}N\}_{\mu,N} \\
&= \{\sqrt{p_{\mathcal{M}}(M)}M\}_M,
\end{aligned} \tag{83}$$

where we group the same Pauli terms and introduce

$$p_{\mathcal{M}}(M) = \sum_{\substack{\mu,N:\\ R_{\partial N+\partial(\mu+\widehat{\mu})}N=M}} p_{\mathcal{R}}(\mu)p_{\mathcal{N}}(N). \tag{84}$$

Since for any stabilizer syndrome σ and Pauli X operator N we have $\Pi_\sigma^{\mathcal{S}}=\sum_{\varphi:\partial\varphi=\sigma}\Pi_\varphi^{\mathcal{G}}$ and $\Pi_\sigma^{\mathcal{S}}N\Pi_0^{\mathcal{S}}=\delta_{\sigma,\partial N}N\Pi_0^{\mathcal{S}}$, where $\delta_{\sigma,\partial N}$ is the Kronecker delta, we obtain

$$\begin{aligned}
&\mathcal{R}\circ\mathcal{N}\circ\Pi_0^{\mathcal{S}} \\
&\quad= \{\sqrt{p_{\mathcal{R}}(\mu)p_{\mathcal{N}}(N)}R_{\partial(\varphi+\mu+\widehat{\mu})}\Pi_\varphi^{\mathcal{G}}N\Pi_0^{\mathcal{S}}\}_{\mu,\varphi,N} \\
&\quad= \{\sqrt{p_{\mathcal{R}}(\mu)p_{\mathcal{N}}(N)}R_{\sigma+\partial(\mu+\widehat{\mu})}\Pi_\sigma^{\mathcal{S}}N\Pi_0^{\mathcal{S}}\}_{\mu,\sigma,N} \\
&\quad= \mathcal{M}\circ\Pi_0^{\mathcal{S}}.
\end{aligned} \tag{85}$$

Thus, proving the theorem is equivalent to showing that $\mathcal{M}\in\mathbb{N}_{\tau',\epsilon'}$.

Let us consider the $\mathcal{R}_0$-dependent channel for $\mathcal{M}$, i.e.,

$$\overline{\mathcal{M}} = \{\sqrt{p_{\mathcal{M}}(M)}R_{\partial M}\}_M = \left\{\sqrt{p_{\overline{\mathcal{M}}}(M')}M'\right\}_{M'}, \tag{86}$$

where we group the same Pauli terms and introduce

$$\begin{aligned}
p_{\overline{\mathcal{M}}}(M') &= \sum_{M:R_{\partial M}=M'} p_{\mathcal{M}}(M) \\
&= \sum_{\substack{\mu,N:\\ R_{\partial(\mu+\widehat{\mu})}=M'}} p_{\mathcal{R}}(\mu)p_{\mathcal{N}}(N) = \sum_{\mu:R_{\partial(\mu+\widehat{\mu})}=M'} p_{\mathcal{R}}(\mu).
\end{aligned} \tag{87}$$

In the second equality above we use Eq. (84) and the fact that for $M=R_{\partial N+\partial(\mu+\widehat{\mu})}N$ we have $\partial M=\partial(\mu+\widehat{\mu})$. By definition, $\overline{\mathcal{M}}$ is $\mathcal{R}_0$-equivalent to $\mathcal{M}$. We now show that $\overline{\mathcal{M}}$ is τ'-bounded by finding for any Pauli X operator M an upper bound on

$$\sum_{M'\supseteq M} p_{\overline{\mathcal{M}}}(M') = \sum_{\mu:R_{\partial(\mu+\widehat{\mu})}\supseteq M} p_{\mathcal{R}}(\mu). \tag{88}$$

We proceed by first proving for any Pauli X operator M the following inequality

$$\begin{aligned}
&\sum_{\mu:R_{\partial(\mu+\widehat{\mu})}\supseteq M} p_{\mathcal{R}}(\mu) \\
&\qquad\leq \sum_{s\geq t|M|}\sum_{W\in\mathfrak{C}(s,M)}\sideset{}{'}\sum_{\mu'\subseteq W:|\mu'|\geq rs}\sum_{\mu\supseteq\mu'} p_{\mathcal{R}}(\mu),
\end{aligned} \tag{89}$$

where $\mathfrak{C}(s,M)$ is the collection of connected covers of M within the hypergraph $G_{\mathcal{L}}$ and $\sum'$ denotes that we only sum over the measurement errors. Let μ be a measurement error, for which $p_{\mathcal{R}}(\mu)$ appears on the left-hand side of the inequality. We now show that the variables s, W and μ' in Eq. (89) can admit the following values

$$W = \left((\mu+\widehat{\mu}) \cup R_{\partial(\mu+\widehat{\mu})}\right)^{\cap M},\ s = |W|,\ \mu' = \mu \cap W. \tag{90}$$

This, in turn, implies that the term $p_{\mathcal{R}}(\mu)$ also appears on the right-hand side and, subsequently, establishes the inequality, as all the terms in the inequality are nonnegative.

Let $\widehat{\mu}' = \widehat{\mu} \cap W$ and $R' = R_{\partial(\mu+\widehat{\mu})} \cap W$. We then have $R' \supseteq M$, $W = (\mu'+\widehat{\mu}') \cup R'$ and $|\mu'+\widehat{\mu}'| = |\mu'| + |\widehat{\mu}'|$. One can also verify that $\mu'+\widehat{\mu}'$ is a flux, as it satisfies the Gauss law, i.e., $\delta_R(\mu'+\widehat{\mu}') = 0$, and that $\mu'+\widehat{\mu}'$ and R' have the same stabilizer syndromes, i.e., $\partial(\mu'+\widehat{\mu}') = \partial R'$. Since $\widehat{\mu}$ is the minimum-weight estimate of the measurement error μ, we have $|\widehat{\mu}'| \leq |\mu'|$; otherwise, $\mu'+\widehat{\mu}'+\widehat{\mu}$ would be an estimate of the measurement error μ of weight smaller than $\widehat{\mu}$. The assumption about confinement of the flux guarantees that for the flux $\mu'+\widehat{\mu}'$ there exists a Pauli X operator N, such that $\partial N = \partial(\mu'+\widehat{\mu}')$ and $|N| \leq c_{\text{flu}}|\mu'+\widehat{\mu}'|$. Since $R_{\partial(\mu+\widehat{\mu})}$ is the minimum-weight recovery operator with the stabilizer syndrome $\partial(\mu+\widehat{\mu})$, we also have $|R'| \leq |N|$; otherwise, $NR'R_{\partial(\mu+\widehat{\mu})}$ would be a recovery operator with the stabilizer syndrome $\partial(\mu+\widehat{\mu})$ of weight smaller than $R_{\partial(\mu+\widehat{\mu})}$. By lower bounding the cardinality of W as follows

$$\begin{aligned} |W| &= |\mu'+\widehat{\mu}'| + |R'| \geq c_{\text{flu}}^{-1}|N| + |R'| \\ &\geq (1+c_{\text{flu}}^{-1})|R'| \geq (1+c_{\text{flu}}^{-1})|M| \end{aligned} \tag{91}$$

we obtain that $s \geq t|M|$. Thus, s specified in Eq. (90) appears in Eq. (89). Since $R_{\partial(\mu+\widehat{\mu})} \supseteq M$, we conclude that W specified in Eq. (90) is a connected cover of M within $G_{\mathcal{L}}$, i.e., $W \in \mathfrak{C}(s,M)$, and appears in Eq. (89). Also, by upper bounding the cardinality of W as follows

$$\begin{aligned} |W| &= |\mu'| + |\widehat{\mu}'| + |R'| \leq 2|\mu'| + |N| \\ &\leq 2|\mu'| + c_{\text{flu}}|\mu'+\widehat{\mu}'| \leq 2(1+c_{\text{flu}})|\mu'| \end{aligned} \tag{92}$$

we obtain that $|\mu'| \geq rs$. Thus, μ' specified in Eq. (90) appears in Eq. (89).

Using the fact that $p_{\mathcal{R}}$ is η-bounded we obtain

$$\begin{aligned} &\sum_{s\geq t|M|} \sum_{W\in\mathfrak{C}(s,M)} \sideset{}{'}\sum_{\mu'\subseteq W:|\mu'|\geq rs} \sum_{\mu\supseteq\mu'} p_{\mathcal{R}}(\mu) \\ &\quad\leq \sum_{s\geq t|M|} \sum_{W\in\mathfrak{C}(s,M)} \sideset{}{'}\sum_{\mu'\subseteq W:|\mu'|\geq rs} \eta^{|\mu'|} \\ &\quad\leq \sum_{s\geq t|M|} \sum_{W\in\mathfrak{C}(s,M)} 2^{|W|}\eta^{rs} \\ &\quad\leq \sum_{s\geq t|M|} (2\eta^r)^s|\mathfrak{C}(s,M)|. \end{aligned} \tag{93}$$

We can then use (connected covers) Lemma 2 to bound the size of $\mathfrak{C}(s,M)$ and obtain

$$\begin{aligned} \sum_{s\geq t|M|} (2\eta^r)^s|\mathfrak{C}(s,M)| &\leq \sum_{s\geq t|M|} (2\eta^r)^s \frac{(\mathfrak{e}z)^s}{\mathfrak{e}z^{|M|}} \\ &= \frac{((2\mathfrak{e}\eta^r)^t z^{t-1})^{|M|}}{\mathfrak{e}(1-2\mathfrak{e}z\eta^r)} \\ &\leq \frac{(2^{2t-1}(\mathfrak{e}\eta^r)^t(\Delta_{\mathcal{L}}-1)^{t-1})^{|M|}}{\mathfrak{e}-4\mathfrak{e}^2(\Delta_{\mathcal{L}}-1)\eta^r} \\ &\leq (2^{2t-1}(\mathfrak{e}\eta^r)^t(\Delta_{\mathcal{L}}-1)^{t-1})^{|M|}, \end{aligned} \tag{94}$$

where we use the upper bound on η and $z = \max_{e\in E_{\mathcal{L}}}\sum_{v\in e}(\deg v - 1) \leq 2(\Delta_{\mathcal{L}}-1)$, as any hyperedge of the hypergraph $G_{\mathcal{L}}$ contains at most two vertices. Thus, $\overline{\mathcal{M}}$ is τ'-bounded and, subsequently, $\mathcal{M} \in \mathbb{N}_{\tau',*}$.

To upper bound the failure probability for $\mathcal{M}$ we first observe that

$$\begin{aligned} \text{fail}(\mathcal{M}) &= \sum_{M:R_{\partial M}M\notin\mathcal{G}} p_{\mathcal{M}}(M) \\ &= \sum_{\substack{\mu,N:\\ R_{\partial(\mu+\widehat{\mu})}R_{\partial N+\partial(\mu+\widehat{\mu})}N\notin\mathcal{G}}} p_{\mathcal{R}}(\mu)p_{\mathcal{N}}(N) \\ &= \text{fail}\left(\left\{\sqrt{p_{\mathcal{R}}(\mu)}R_{\partial(\mu+\widehat{\mu})}\right\}_\mu \circ \mathcal{N}\right) \\ &= \text{fail}(\overline{\mathcal{M}}\circ\mathcal{N}). \end{aligned} \tag{95}$$

Let $\mathcal{N}' = \{\sqrt{p_{\mathcal{N}'}(N')}N'\}_{N'}$ be a τ-bounded Pauli X channel, which is $\mathcal{R}_0$-equivalent to $\mathcal{N}$. Then, (composition) Lemma 5 implies that the composite channel $\overline{\mathcal{M}}\circ\mathcal{N}'$ is $(\tau+\tau')$-bounded. Using the inequalities from Lemma 4 and applying (decoding failure) Lemma 3 to $\mathcal{N}'$ and $\overline{\mathcal{M}}\circ\mathcal{N}'$ we obtain

$$\begin{aligned} &\text{fail}\left(\overline{\mathcal{M}}\circ\mathcal{N}\right) \leq \text{fail}(\overline{\mathcal{M}}) + \text{fail}(\mathcal{N}) + \text{fail}\left(\overline{\overline{\mathcal{M}}\circ\overline{\mathcal{N}}}\right) \\ &\quad= \text{fail}(\mathcal{N}) + \text{fail}\left(\overline{\overline{\mathcal{M}}\circ\overline{\mathcal{N}'}}\right) \\ &\quad\leq \epsilon + \text{fail}(\overline{\mathcal{M}}) + \text{fail}(\mathcal{N}') + \text{fail}\left(\overline{\mathcal{M}}\circ\mathcal{N}'\right) \\ &\quad\leq \epsilon + f(\tau) + f(\tau+\tau'). \end{aligned} \tag{96}$$

Thus, $\text{fail}(\mathcal{M}) \leq \epsilon + f(\tau) + f(\tau+\tau')$ and subsequently $\mathcal{M} \in \mathbb{N}_{*,\epsilon'}$. □

Using (single-shot QEC) Theorem 1 and (composition) Lemma 5 we also conclude that after n repeated rounds of Pauli X noise and single-shot MWPM decoding channel we have

$$(\mathbb{R}_\eta \circ \mathbb{N}_{\tau,\epsilon})^n \circ \Pi_0^{\mathcal{S}} \subseteq \mathbb{N}_{\tau',\epsilon(n)} \circ \Pi_0^{\mathcal{S}}, \tag{97}$$

where $\epsilon(n) = n\epsilon + nf(\tau) + (n-1)f(\tau') + (2n-1)f(\tau+\tau') + (n-1)f(\tau+2\tau')$, provided that $\tau+2\tau' < (2(\Delta_{\text{qub}}-1))^{-2}$. Thus, the residual noise in the 3D STC does not accumulate in an uncontrollable way after we perform multiple rounds of single-shot QEC, and the logical information encoded in the 3D STC will be protected for a long time.

SUPPLEMENTARY REFERENCES

[1] E. Dennis, A. Kitaev, A. Landahl, and J. Preskill, Topological quantum memory, Journal of Mathematical Physics **43**, 4452 (2002).

[2] S. Bravyi, G. Duclos-Cianci, D. Poulin, and M. Suchara, Subsystem surface codes with three-qubit check operators, Quantum Information and Computation **13**, 963 (2013).

[3] M. B. Hastings and X.-G. Wen, Quasiadiabatic continuation of quantum states: The stability of topological ground-state degeneracy and emergent gauge invariance, Physical Review B **72**, 045141 (2005).

[4] S. Bravyi, M. B. Hastings, and F. Verstraete, Lieb-Robinson bounds and the generation of correlations and topological quantum order, Physical Review Letters **97**, 1 (2006).

[5] X. Chen, Z.-C. Gu, and X.-G. Wen, Local unitary transformation, long-range quantum entanglement, wave function renormalization, and topological order, Phys. Rev. B **82**, 155138 (2010).

[6] A. Kubica and M. E. Beverland, Universal transversal gates with color codes: A simplified approach, Physical Review A **91**, 032330 (2015).

[7] T. Jochym-O'Connor and T. J. Yoder, Four-dimensional toric code with non-Clifford transversal gates, Physical Review Research **3**, 13118 (2021).

[8] H. Bombín, Single-Shot Fault-Tolerant Quantum Error Correction, Physical Review X **5**, 031043 (2015).

[9] P. Aliferis, D. Gottesman, and J. Preskill, Accuracy threshold for postselected quantum computation, Quantum Information and Computation **8**, 181 (2008).

[10] A. A. Kovalev and L. P. Pryadko, Fault tolerance of quantum low-density parity check codes with sublinear distance scaling, Physical Review A **87**, 020304 (2013).

[11] D. Gottesman, Fault-Tolerant Quantum Computation with Constant Overhead, arXiv:1310.2984 (2013).
